# The catalytic activity of the translation termination factor methyltransferase Mtq2-Trm112 complex is required for large ribosomal subunit biogenesis

**DOI:** 10.1093/nar/gkaa972

**Published:** 2020-11-09

**Authors:** Caroline Lacoux, Ludivine Wacheul, Kritika Saraf, Nicolas Pythoud, Emmeline Huvelle, Sabine Figaro, Marc Graille, Christine Carapito, Denis L J Lafontaine, Valérie Heurgué-Hamard

**Affiliations:** UMR8261 (CNRS, Université de Paris), Institut de Biologie Physico-Chimique, 13 rue Pierre et Marie Curie, 75005 Paris, France; RNA Molecular Biology, Fonds de la Recherche Scientifique (F.R.S.-FNRS), Université Libre de Bruxelles Cancer Research Center (U-CRC), Center for Microscopy and Molecular Imaging (CMMI), Gosselies, Belgium; RNA Molecular Biology, Fonds de la Recherche Scientifique (F.R.S.-FNRS), Université Libre de Bruxelles Cancer Research Center (U-CRC), Center for Microscopy and Molecular Imaging (CMMI), Gosselies, Belgium; Laboratoire de Spectrométrie de Masse Bio-Organique (LSMBO), UMR 7178, IPHC, Université de Strasbourg, CNRS, Strasbourg, France; UMR8261 (CNRS, Université de Paris), Institut de Biologie Physico-Chimique, 13 rue Pierre et Marie Curie, 75005 Paris, France; UMR8261 (CNRS, Université de Paris), Institut de Biologie Physico-Chimique, 13 rue Pierre et Marie Curie, 75005 Paris, France; Laboratoire de Biologie Structurale de la Cellule (BIOC), CNRS, Ecole polytechnique, Institut Polytechnique de Paris, F-91128 Palaiseau, France; Laboratoire de Spectrométrie de Masse Bio-Organique (LSMBO), UMR 7178, IPHC, Université de Strasbourg, CNRS, Strasbourg, France; RNA Molecular Biology, Fonds de la Recherche Scientifique (F.R.S.-FNRS), Université Libre de Bruxelles Cancer Research Center (U-CRC), Center for Microscopy and Molecular Imaging (CMMI), Gosselies, Belgium; UMR8261 (CNRS, Université de Paris), Institut de Biologie Physico-Chimique, 13 rue Pierre et Marie Curie, 75005 Paris, France

## Abstract

The Mtq2-Trm112 methyltransferase modifies the eukaryotic translation termination factor eRF1 on the glutamine side chain of a universally conserved GGQ motif that is essential for release of newly synthesized peptides. Although this modification is found in the three domains of life, its exact role in eukaryotes remains unknown. As the deletion of *MTQ2* leads to severe growth impairment in yeast, we have investigated its role further and tested its putative involvement in ribosome biogenesis. We found that Mtq2 is associated with nuclear 60S subunit precursors, and we demonstrate that its catalytic activity is required for nucleolar release of pre-60S and for efficient production of mature 5.8S and 25S rRNAs. Thus, we identify Mtq2 as a novel ribosome assembly factor important for large ribosomal subunit formation. We propose that Mtq2-Trm112 might modify eRF1 in the nucleus as part of a quality control mechanism aimed at proof-reading the peptidyl transferase center, where it will subsequently bind during translation termination.

## INTRODUCTION

In the three domains of life, cellular components are targeted by numerous post-transcriptional and post-translational modifications. Methylation is one of the most common modifications. It occurs extensively on the translational apparatus, and ribosomal RNA (rRNA), transfer RNA (tRNA), messenger RNA (mRNA), ribosomal proteins (r-proteins) and translational factors are all modified (1–7). In the yeast *Saccharomyces cerevisiae*, two-thirds of the known methyltransferases are targeting substrates involved in translation and many of these enzymes are conserved in human (8). Methylation is mainly catalyzed by *S*-adenosyl-l-methionine (SAM)–dependent methyltransferases (8,9). However, in most cases, the role of the modification enzymes, and the grafted methyl groups, are not known.

Most rRNA modifications, including 2′-*O* ribose methylation, pseudouridylation, base methylation, acetylation and aminocarboxypropylation are found on residues clustered around the functional centers of the ribosomal subunits (10). They are assumed to be important for accurate and efficient translation, although in most cases this remains to be demonstrated. In budding yeast, the entire set of rRNA base methyltransferases has been identified, and many of them are important for the synthesis of ribosomal subunits (3). However, quite remarkably, it is the proteins themselves, rather than their catalytic activity, that are important for those involved in 40S subunit production (11–14).

In addition to rRNAs, r-proteins are also methylated on different amino acid residues (15). R-protein methyltransferases are not essential for cell growth in yeast, at least under optimal laboratory conditions. However, null mutants are less competitive for growth in fitness assays. At least half of the r-protein methyltransferases play a role in ribosome biogenesis, as judged by altered 60S-to-40S subunit ratios (8). In addition, translational fidelity assays demonstrated that all the r-protein methyltransferases tested are important for translation accuracy at the elongation and/or termination steps (5,8,16). Up until now, it is not clear whether it is the catalytic activity of the r-protein methyltransferase or its presence in maturing subunits that is important for ribosomal subunit production. Additionally, ribosomal assembly factors may also be subjected to post-translational modifications, but this area remains largely unexplored.

Translational factors, such as elongation factors (eEF1A, eEF2, eEF3 in yeast) and termination factors (RF1 and RF2, in bacteria; aRF1 in archaea; eRF1 and mitochondrial RF1, in eukaryotes) are also methylated, and to date ten specific methyltransferases (Mtq1, Mtq2, Efm1-7 and Dph5) have been identified in yeast (1,6,16–19).

On translation termination factors, the glutamine side chain of the universally conserved GGQ motif, which interacts with the ribosomal peptidyl transferase center (PTC) to catalyze the release of the new protein, is methylated in the three domains of life. Several observations argue in favor of a biologically important role for this evolutionarily conserved GGQ motif modification (1,6,20–23).

One such observation is that cells have evolved convergent machineries to methylate the glutamine side chain of their GGQ motifs despite the unrelated structures of bacterial and eukaryal/archaeal termination factors. Indeed, while bacterial RF1 and RF2 are methylated by a single-chain protein named PrmC, archaeal and eukaryal termination factor methylation relies on a heterodimeric complex consisting of the Mtq2 (HEMK2, in human cells) catalytic subunit and its co-activator Trm112 (TRMT112, in human) (23–26). Trm112 interacts with and activates three other methyltransferases, which all modify substrates involved in translation. These methyltransferases are Bud23, which catalyzes the N^7^ methylation of 18S rRNA base G_1575_, and Trm9 and Trm11, which methylate different positions on specific tRNAs (27,28). Trm112 is important for the metabolic stability of Mtq2 and Bud23, and possibly other methyltransferases (14,27,29). Another observation is that *Escherichia coli prmC* inactivation affects cell growth, and loss of release factor methylation decreases the catalytic efficiency of the termination reaction as well as the affinity of RF2 for ribosomes, at least *in vitro* (30,31). The methylation also increases the efficiency of termination *in vivo* (32).

Physiological studies conducted on archaeal cells in which the *mtq2* gene was deleted, and in human cells depleted of HEMK2, demonstrated that the protein is required for optimal cell growth (23,33). Depletion of the murine MTQ2 ortholog (mN6amt1, also called PRED28) also leads to defects in cell proliferation and to early embryonic death, which is typically observed with ribosome biogenesis inhibitions (33–35).

In *S. cerevisiae*, the deletion of the *MTQ2* gene leads to slow growth on rich media and to increased sensitivity towards aminoglycoside antibiotics, such as paromomycin and geneticin (6), which are known to affect translation fidelity through ribosome binding (6). This suggested that eRF1 methylation might be important for translation termination; however, this does not seem to be the case as translation termination efficiency is not detectably affected in *mtq2*Δ cells (5). Therefore, the exact role of eRF1 methylation remains unknown.

Here, we report an unexpected function of Mtq2 in ribosome biogenesis in budding yeast. Specifically, we show that Mtq2 is associated with large ribosomal subunit precursors, and that its catalytic activity is required for pre-60S nucleolar release and efficient production of 5.8S and 25S rRNAs.

## MATERIALS AND METHODS

### Bacterial and yeast strains

*Escherichia coli* DH5α was used for DNA amplification and cloning purposes. Antibiotics were added, when required, at the final concentrations of 50 μg/ml for kanamycin, 200 μg/ml for ampicillin and 15 μg/ml for chloramphenicol. Yeast strains were either purchased from Euroscarf, or made by the transformation and integration on the chromosome, by homologous recombination, of PCR cassettes generated with specific DNA templates. The oligonucleotides used are listed in Supplementary Table S1. Yeast strains used in this study are listed in Supplementary Materials and MethodsSupplementary Table S2. Cells were grown at 30°C unless otherwise stated. Cells were either grown in complete yeast extract-peptone-dextrose (2% each) (YPD) or in synthetic minimal medium, supplemented with 2% of glucose. Paromomycin was added to a final concentration of 800 μg/ml and cycloheximide to a final concentration of 100 μg/ml. G418 and NAT were added to a final concentration of 400 μg/ml and 60 μg/ml, respectively. In all experiments, we have used the *mtq2*Δ*::kanMX* strain from Euroscarf, except for panel F in the first figure (where we have used *mtq2*Δ*::HIS3* and an isogenic control). The *mtq2*Δ*::HIS3* and *mtq2*Δ*::kanMX* have exactly the same doubling time.

**Figure 1.**
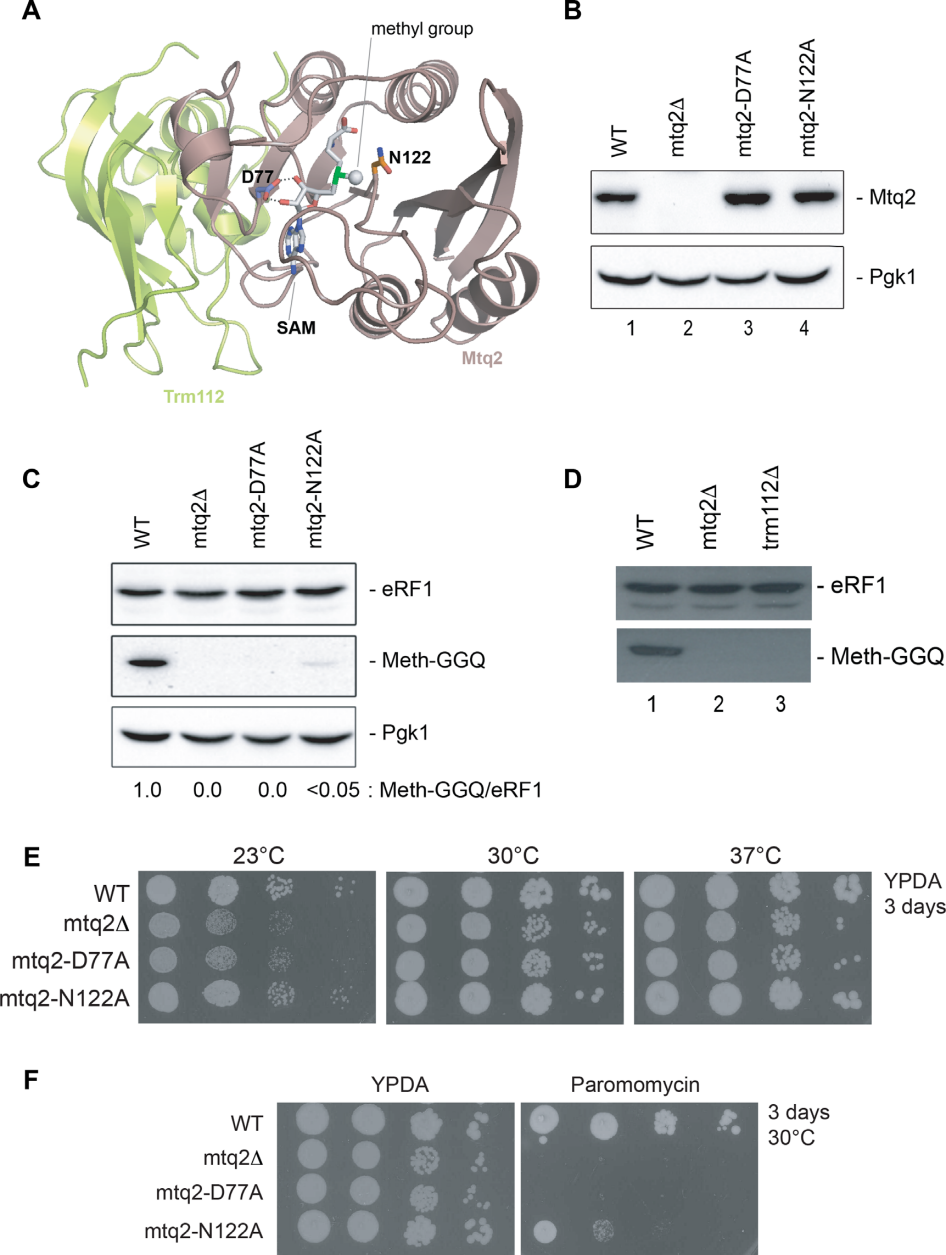
The methylation activity of Mtq2 is important for cell growth and antibiotic resistance. (**A**) Ribbon representation of the crystal structure of the *E. cuniculi* Mtq2-Trm112 methyltransferase complex. The side chains corresponding to the D77 and N122 residues mutated in this study are shown as sticks. The *S*-adenosyl methionine (SAM), which donates the methyl group, is shown in grey sticks. Hydrogen bonds formed between D77 side chain and the ribose hydroxyl groups of the SAM are depicted by black dashed lines. Gray sphere, methyl group of SAM. (**B**) Mtq2-D77A and Mtq2-N122A are metabolically stable. Equal amounts of total protein extracts from the indicated yeast strains grown at 30°C were analyzed by western blotting with antibodies specific to Mtq2. The blot was probed for Pgk1 as a control for loading. (**C**) eRF1 is not methylated in cells expressing Mtq2 D77A or N122A mutants. The western blots were probed for total eRF1 or the methylated form of eRF1 (Meth-GGQ). A residual level of <5% eRF1 methylation is observed in *mtq2-N122A* cells. The blot was probed for Pgk1 as a control for loading. (**D**) Trm112 is required for eRF1 methylation in cells. Western blot analysis as described in panel C. (**E**) The methylation function of Mtq2 is important for cell growth. Serial dilutions of yeast cells of the indicated strains were spotted on agar plates containing glucose-rich-based medium (YPDA) and incubated at the indicated temperatures for 3 days. From Left to right, 10× dilutions. See complementary Supplementary Table S4 for doubling times. (**F**) The methylation function of Mtq2 is important for antibiotic resistance. Growth assay on agar plate, as described in panel E, in the presence of paromomycin (800 μg/ml). Plates incubated at 30°C for 3 days.

### MTQ2 gene tagging, generation of mtq2 mutants and TAP-tagged yeast strains

3′ tagging of *MTQ2* chromosomal locus was performed by homologous gene recombination in strain BY4741 using the selectable marker His3MX6 and the ADH1 transcription terminator from plasmid pFA6a-GFP(S65T)-His3MX6 (36). These elements were amplified together with oligonucleotides Mtq2F3up and Mtq2R1low as listed in Supplementary Table S1. yVH192 was recovered on HC-His plates, and the correct integration of the marker cassette was confirmed by PCR and sequencing.

Genomic DNA from strain yVH192 was then used for a PCR reaction with the appropriate oligonucleotide that introduced the desired point mutation in tandem with oligonucleotide Mtq2R1low; amplification with primer Mtq2D77A and Mtq2R1low was performed to produce the yVH200 strain carrying the D77A mutation in *mtq2*. Then, the purified PCR product was used to transform BY4741. The presence of the mutation in candidate colonies was verified by restriction-fragment-length polymorphism and DNA sequencing. This two-step mutagenesis method was described by Toulmay and Schneiter (37), and we previously showed its general applicability and efficiency (14,38). The FF12 strain deleted for the *MTQ2* gene (*mtq2*Δ::*his3)* strain was described previously (26). TAP-tagged yeast strains used in this study were constructed as described previously (39). Briefly, strains yVH200 (*mtq2-D77A*) and yVH201 (*mtq2-N122A*) yeast constructs were transformed by a DNA fragment containing the sequence encoding the TAP tag and the *Kluyveromyces lactis URA3* sequence that could recombine upstream of the chromosomal *MTQ2* stop codon of the genomic *MTQ2* copy. The DNA fragment was amplified from the pBS1539 plasmid using the Down tap Mtq2 and Up tap Mtq2 oligonucleotides. Ura+ transformants were then selected on HC-Ura selective plates. Strains CL3 and CL5 were verified by PCR, sequencing and western blot using both anti-Mtq2 and anti-TAP tag antibodies. The wild-type Mtq2-TAP strain was purchased from Open Biosystem.

For the TAP-alone control strain, a TAP cassette was integrated at the *BAT2* locus by homologous recombination, resulting in the expression of a stable full-length TAP polypeptide directly under the control of the *BAT2* constitutive promoter (strain YDL2618). *BAT2* is a metabolic gene that encodes an enzyme involved in the last step of leucine formation. Genome-wide studies estimated that Bat2 is expressed at ∼25 900 copies/cell (40), which is far more than most ribosome synthesis factors, and this makes it a suitable control for nonspecific interactions.

The Nop2-TAP, Nog2-TAP and Nmd3-TAP strains were constructed in the Mtq2-N122A background in two steps. The selectable marker His3MX6 from yVH201 was changed into kanMX6 by gene recombination using the kanMX6 marker and *ADH* terminator from plasmid pFA6-GFP(S65T)-kanMX6. Amplification was done with oligonucleotides Mtq2F3up and mtq2R1low. yVH440 was recovered on YPDA-G418 plates. Mtq2N122A linked to kanMX6 chromosomal locus was then mobilized into the three TAP strains by recombination with PCR products obtained with oligonucleotides Mtq2kan571 and Mtq2kan2977 that are, respectively, upstream and downstream of Mtq2kanMX6 locus. Correct integration of the Mtq2N122 mutation was confirmed by PCR and sequencing.

Deletion of *MTQ2* with the kanMX cassette in the *trf4* and *rrp6* mutant backgrounds was performed by homologous recombination. Knock-out genes were amplified from appropriate strains with oligonucleotides indicated in Supplementary Table S1 and transformed in the Y04074 (*mtq2*Δ*::kanMX*) strain. Selection was done either on HC-His plates or YPDA-NAT plates.

For localization of Mtq2 by fluorescence microcopy, we used plasmid pVH511 expressing Mtq2 fused to eGFP on its C-terminus and under the control of *ADH* promoter and *CYC1* terminator. Mtq2 was amplified from the wild-type yeast strain BY4741 with oligonucleotides Mtq2BamHI-GFPpRS5′ and Mtq2XhoI-GFPpRS3′ introducing respectively *BamH*I and *Xho*I restriction sites. After digestion, this fragment was inserted into a derivative of the p415 plasmid carrying an *ADH* promoter and a polylinker upstream of the gene encoding eGFP (41). pVH512 expressing eRF1 fused to eGFP was made by the same method. The *SUP45* gene encoding eRF1, was amplified with oligonucleotides eRF1XbaI-GFPpRS5′ and eRF1XhoI-GFPpRS3′ and cloned into the same p415 derivative.

### Western blot analysis

Proteins were separated on 10% or 12% SDS-polyacrylamide gels, transferred to nitrocellulose membranes (Ambion), and western blot analyses were performed with specific primary antibodies and peroxidase-coupled secondary antibodies (Sigma). We used the Clarity chemiluminescent substrate (Biorad) and Biorad Chemidoc XRS camera to visualize the blot. Quantification was done using Image lab software (Biorad). We used anti-Mtq2-Trm112 and eRF1 antibodies raised in rabbit against purified recombinant yeast Mtq2-Trm112 complex and recombinant eRF1, respectively. Mouse monoclonal anti-PGK1 was purchased from Invitrogen. Antibodies against methylated eRF1 were raised in rabbit against the peptide HGRGG**Q**SALRFA present in yeast eRF1, where the glutamine residue is N5-monomethylated (Eurogentec). These antibodies were then purified based on their retention on a column grafted with the methylated peptide but not on a column grafted with the unmethylated peptide.

### RNA extraction, northern blot, quantitation

RNA extraction from yeast cells and northern blots were performed as described previously (42). Oligonucleotides used in the hybridizations are listed in Supplementary Materials and MethodsSupplementary Table S3. For the analysis of high molecular weight species, 10 μg of total RNA was separated on 1.2% agarose – 6% formaldehyde gels; for low molecular weight species, 5 μg of total RNA was separated on 8% urea–acrylamide gels. Phosphor imager quantification used a Fuji FLA-7000 and the native Multi Gauge software (v3.1).

### Fluorescence microscopy

Microscopy was performed on a Zeiss Axio Observer Z1 microscope driven by MetaMorph (MDS Analytical Technologies, Canada). Images were captured in widefield or in confocal mode (using a Yokogawa spindisk head) and the HQ2 camera with a laser illuminator from Roper (405 nm 100 mW Vortran, 491 nm 50 mW Cobolt Calypso, and 561 nm 50 mW Cobolt Jive) and 63× or 100× objectives (Zeiss).

### Polysome analysis

Yeast cells were grown to an OD_600 nm_ of ∼0.5 in YPDA (or 2% sugar-based selective synthetic minimal medium). Cycloheximide was then added to a final concentration of 100 μg/ml. Extracts were prepared in a buffer containing 10 mM Tris–HCl (pH 7.4), 100 mM NaCl, 30 mM MgCl_2_ and 50 μg/ml cycloheximide by vortexing in the presence of glass beads. Extracts were fractioned by ultracentrifugation on a sucrose gradient (10–50%) in a buffer containing 50 mM Tris–HCl (pH 7.4), 12 mM MgCl_2_, 50 mM NH_4_Cl and 1 mM DTT for 2h45 min at 39 000 rpm at 4°C (SW41 rotor). The fractions were recovered with an ISCO fractionator, and the absorbance at 254 nm was measured. The ratio between the small and the large subunits was obtained by analyzing the absorbance profiles of whole cellular extracts prepared in the absence of MgCl_2_. When needed, proteins from each fraction were precipitated with TCA and sodium deoxycholate.

### Affinity purification

An affinity purification protocol optimized to detect transient interactions was used (43–45). Briefly, yeast cells were collected, suspended in resuspension buffer, frozen in liquid nitrogen and then lysed in solid phase by cryo-milling on a MM 400 mixer (Retsch's) and stored at −80°C until needed. The resuspension buffer consisted of: 1.2% PVP-40, 20 mM HEPES pH 7.4, 1:100 Sigma protease inhibitor cocktail, and 1:100 solution P (2 mg pepstatin A and 90 mg PMSF in 5 ml ethanol) and 1 mM DTT. The extract was resuspended in RNP buffer (20 mM HEPES pH 7.4, 110 mM KOAc, 0.5% Triton X-100, 0.1% Tween-20, 1:5000 Ambion SuperRNAsin, 1:5000 Sigma antifoam, 1:100 solution P, 150 mM NaCl) and incubated with prepared magnetic beads with rabbit IgG (Dynal) for 1 h at 4°C. The beads were collected using a magnet, washed five times, and eluted twice for 20 minutes at RT under denaturing conditions (500 mM NH_4_OH/0.5 mM EDTA). Eluates were then lyophilized for LC–MS/MS analysis. For coated magnetic beads preparation, M280 Dynabeads Sheep anti-rabbit IgG (Dynal, Invitrogen) were saturated with rabbit IgG antibodies (Sigma) for 1 h at +4°C as recommended by the manufacturer. Magnetic beads were then washed extensively and rabbit IgG cross-linked using glutaraldehyde as described previously (45).

### Mass spectrometry experiments and data analysis

These different steps are described in details in Supplementary data. Briefly, proteins were concentrated by SDS-PAGE gel and then digested with trypsin while in the gel. Peptides were analyzed by NanoLC-MS/MS. Data were processed using MaxQuant including Andromeda search engine. Label Free Quantification intensities were calculated. The dataset was deposited on the ProteomeXchange Consortium severe via the PRIDE partner repository with the dataset identifier PXD018733 (46).

### Statistical data analysis

Statistical analyses were performed using ProStaR software suite (47–49). Proteins identified in the reverse and contaminant databases, proteins only identified by site, and proteins for which less than two LFQ values were available in a single condition were removed from the list. After log_2_ transformation, imputation of missing values was performed. For each sample, the slsa algorithm was used for the POV (Partially Observed Values) imputation, and missing values were replaced by the 2.5 percentile value for the MEC (Missing on the Entire Condition); statistical testing was performed using Welch's t-test. Slim (sliding linear model) (50) method was used to adjust p-values for multiple testing and differentially expressed proteins were sorted out using a p-value threshold that guarantees a FDR below 5%.

### *In vivo* labelling of pre-rRNAs (pulse-chase)

*mtq2*Δ and isogenic control cells (transformed with a URA3-expressing plasmid) were grown to mid log phase (OD_600_ of 0.5) in minimal medium lacking uracil. 5 ml of cell culture was labeled with 300 μl uracil (5,6-^3^H) (Perkin Elmer, NET368005MC, 5 mCi/5 ml) for 1 min and then chased with an excess of cold uracil (500 μl at 2.4 mg/ml) prior to collection; 1 ml cell culture aliquots at 0, 1, 2, 5, 10 and 20 min time points were collected and frozen in liquid nitrogen. Total RNA was extracted, separated on a denaturing agarose gel, and transferred to a Genescreen membrane (PerkinElmer, NEF1017001PK). The membrane was exposed to tritium-sensitive Fuji imaging plate (Fuji, BAS TR2025) for 2 weeks. The signal was acquired on a Fuji phosphor imager (Fuji, FLA-7000) with the image reader for FLA-7000 set with default parameters (sensitivity S10000, pixel size 25 μm).

### Primer extension

5 μg total RNA was engaged in a primer extension assay using oligonucleotide LD915 to detect processing site A_3_, as described in (51).

## RESULTS

### The methylation activity of Mtq2 is required for optimal cell growth and antibiotic resistance

To characterize in more details the importance of Mtq2 for cell growth, we made use of an haploid yeast strain (BY4741) in which the open reading frame of the *MTQ2* gene was precisely deleted (*mtq2*Δ, obtained from Euroscarf). The absence of Mtq2 production was confirmed by western-blotting (Figure 1B, lane 2). In addition, to distinguish the effects of loss of Mtq2 function from those of the absence of the protein in cells, we produced two strains expressing a catalytically defective allele known to severely inhibit eRF1 methylation *in vitro* (38). We choose to introduce either the D77A or the N122A mutation. D77 interacts directly with the methyl group donor of SAM, and N122 is part of the enzyme active site signature involved in the recognition of the glutamine side chain to be modified (Figure 1A). The catalytic mutations were generated directly on the chromosome at the endogenous *MTQ2* locus and are thus expressed under the control of the native *MTQ2* promoter. Both mutant proteins (Mtq2-D77A and Mtq2-N122A) were shown to be metabolically stable and expressed at the same level as the wild-type protein, both at the physiological temperature of 30°C (Figure 1B, lanes 3–4) and at the lower temperature of 23°C (data not shown). In agreement with *in vitro* enzymatic assays (38), the two catalytic mutants were largely unable to methylate eRF1 *in vivo*, as shown by differential western blotting analysis using antibodies that recognized either total eRF1 or its methylated form (Figure 1C). In cells expressing the *mtq2-D77A* mutation, the methylation of eRF1 was totally abrogated similarly to what was seen in *mtq2*Δ cells. In cells expressing the *mtq2-N122A* mutation, we observed a minor residual level of eRF1 methylation, which was estimated to be <5% of wild-type levels (Figure 1C). Although the coactivator Trm112 is known to be required for the metabolic stability of Mtq2 and for its activation *in vitro* (25,29), the requirement of Trm112 for eRF1 methylation had not been previously tested directly in cells. We found that in the absence of Trm112 (*trm112*Δ cells), eRF1 was indeed not modified *in vivo* (Figure 1D, lane 3). Interestingly, this is in contrast to the situation that prevails in the archaeon *H. volcanii* where only Mtq2, not Trm112, is required for aRF1 methylation *in vivo* (23).

Mtq2 is known to be important for optimal cell growth (1,6,26). In order to test if it was the protein or its catalytic activity that was important, we performed a plate assay and doubling-time measurements on rich medium (YPDA) at several different temperatures (23, 30 and 37°C). By comparison to the isogenic wild-type control, *mtq2*Δ cells and catalytic mutants were impaired for growth at 30°C, with the D77A mutant being more impacted than the N122 (Figure 1E). Growth measurements in liquid medium showed that the N122A mutant was cryo-sensitive (with a more elevated increase in doubling time at 23°C than 30°C, Supplementary Table S4). In contrast, at 37°C the N122A mutant was unaffected.

*mtq2*Δ cells are known to be sensitive to the aminoglycoside paromomycin ((6) and Figure 1F). Paromomycin binds to helix 44 (decoding site) of 18S rRNA in the 80S ribosome from *S. cerevisiae* (52), promoting missense errors (53,54) and suppression of nonsense mutations (55). We found that the two catalytic mutants were also sensitive to paromomycin (Figure 1F).

In conclusion, while Mtq2 is known to be important for cell growth and resistance towards antibiotics that target translation, it was not clear if its catalytic activity was involved. We have clarified this and shown that the two catalytic mutants strongly affect growth and paromomycin sensitivity. While the D77A mutant perfectly phenocopies the *mtq2*Δ cells, the N122A is slightly less affected for growth (Figure 1E, Supplementary Table S4) and more resistant to paromomycin (Figure 1F). It was likely that the 5% residual methylation activity observed in the N122A strain (see Figure 1C) explained these differences. Note that increased sensitivity towards aminoglycoside antibiotics has also been associated with ribosomal biogenesis defects (e.g. (56)).

### The methylation activity of Mtq2 is required for large ribosomal subunit accumulation

**Figure 2.**
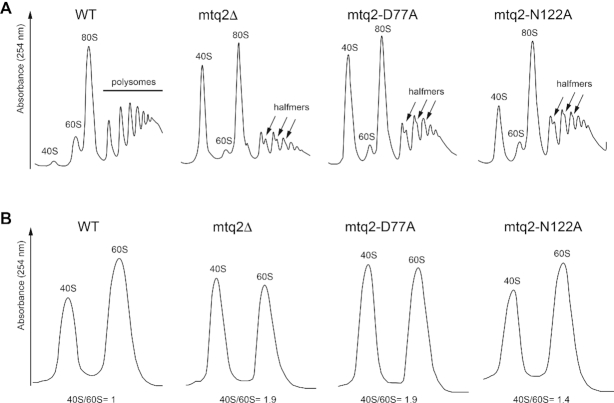
The methylation activity of Mtq2 is required for large ribosomal subunit accumulation. (**A**) Polysomal analysis. Total extracts from the indicated strains were layered on sucrose gradients, separated by velocity centrifugation, and analyzed by monitoring the absorbance at 254 nm. The position of the mature subunits (40S and 60S), monosomes (80S), and polysomes is highlighted. Arrows point to halfmers. (**B**) Ribosomal subunit quantification. Cell extracts from the indicated strains were analyzed by velocity centrifugation in ribosomal subunit dissociation conditions (no Mg^2+^). The 40S/60S subunit ratio was normalized to 1 in wild-type cells.

Mtq2 modifies the translational termination factor eRF1 but its role in translation remains unclear. To explore why its deletion, or catalytic inactivation, conferred slow-growth and increased sensitivity towards antibiotics, we addressed the global translation by performing polysomal analysis on sucrose gradients (Figure 2).

Polysomal analysis allowed us to resolve the 40S, 60S, monosomes (80S), and polysomes according to their size. Briefly, cells were treated with a low concentration of cycloheximide to ‘freeze’ translating ribosomes onto their mRNAs. Cell extracts were then separated on sucrose gradients, and collected with continuous absorbance monitoring at OD_254_. The relative abundance of each peak indicated the efficiency of translation and also provided additional information on ribosomal subunit biogenesis.

While we did observe reduced polysomes in the *mtq2* mutants, the more important differences were totally unexpected: namely, a large increase of free 40S, a concomitant decrease of 60S, and the appearance of ‘halfmers’ (Figure 2, arrows). Halfmers are polysomes bound by an extra 40S subunit which has not been joined by a 60S subunit. Formation of halfmers typically results from 60S ribosomal subunit biogenesis defects (57,58).

The polysomal analysis was a first indication for a ribosomal subunit imbalance in the *mtq2* mutants. To directly prove this was indeed the case, we repeated the experiment under conditions where ribosomal subunits were dissociated by omitting Mg^2+^ in the buffer. This allowed us to precisely quantify the respective amounts of mature 40S and 60S ribosomal subunits (Figure 2B). While in wild-type cells, the 40S-to-60S ratio was of 1.0, as both subunits are produced in equimolar amounts, this ratio was increased to 1.9 in *mtq2*Δ and mtq2-D77A mutants (Figure 2B). The N122A mutant was less affected (ratio of 1.4), likely reflecting the 5% residual eRF1 methylation activity *in vivo* as mentioned above (Figure 1C, and see also growth). To our knowledge, this is the first evidence that the Mtq2 protein and its catalytic activity are involved in 60S subunit biogenesis.

### Mtq2 is associated with large ribosomal subunit precursors

Ribosome biogenesis is a rather complicated process initiated in the nucleolus where nanoscale precursor ribosomal subunits are formed containing ribosomal RNAs, ribosomal proteins, and a myriad of assembly factors that aid numerous RNA folding, processing, modification and transport reactions (59). A large group of over 200 ribosome assembly factors has been identified in yeast (60), and their dynamics of association/dissociation with successive forms of maturing precursor ribosomes established (61).

In order to understand if Mtq2 was directly involved in large ribosomal subunit biogenesis, we tested its physical association with precursor ribosomes by affinity purification of Mtq2 from cells followed by identification of interacting proteins by quantitative proteomics.

Mtq2 was epitope-tagged at its carboxyl terminal end with a tandem high affinity purification tag (TAP-tag, see Materials and Methods). The TAP-tag was inserted at the endogenous *MTQ2* locus directly by homologous recombination in haploid yeast cells and was shown to have no influence on cell growth (data not shown). As a control for specificity, yeast cells expressing only the TAP-tag were used. In our purifications, we only used the Protein A moiety of the TAP tag. In order to achieve statistical validation, the affinity purifications were performed in triplicate. Furthermore, as catalytically-dead proteins are known to sometimes remain bound to their substrates for extended periods of time, we chose to perform the same experiment with the D77A and N122A forms of the protein. Such strategy might allow us to capture transient interactions that would otherwise escape identification.

**Figure 3.**
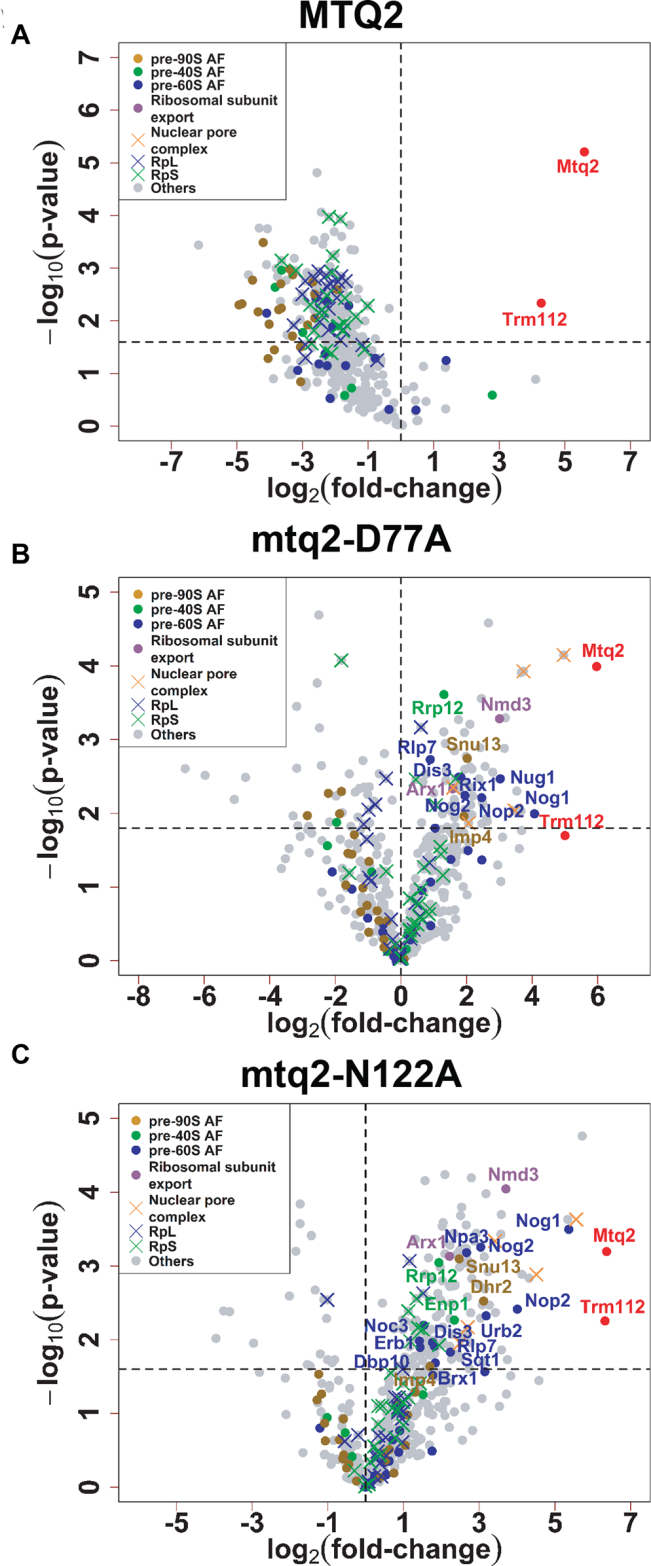
Mtq2 is associated with large ribosomal subunit precursors. The interactome of Mtq2 was established by affinity purification followed by LC-MS/MS identification of the associated proteins. Yeast cells expressing the wild-type (panel **A**) or mutant forms of Mtq2 (panels **B**, for mtq2-D77A and panel **C**, for mtq2-N122A) fused to a high affinity tag were used. The tag was inserted at the carboxyl terminal end of the Mtq2 open reading frame in a haploid yeast strain directly by homologous recombination resulting in a fusion construct expressed from the native Mtq2 promoter. The tagged constructs do not affect cell growth, indicating they are functional. Proteins showing a *P*-value that guarantees an FDR < 0.05 are considered significantly enriched in cells expressing the bait versus the TAP-alone control construct. Table 1 and Supplementary Table S5 contain detailed information on enriched proteins (number of identified peptides, p-values and fold-changes). Interacting proteins are color-coded according to their involvement in specific steps of ribosome biogenesis (assembly of pre-90S, pre-60S, or pre-40S; ribosomal subunit export; nuclear pore complex components) or whether they are core proteins of the small (RpS) or large (RpL) ribosomal subunits.

The resulting three Mtq2 interactomes are displayed as volcano plots in Figure 3. The volcano plots display only the proteins for which a specific enrichment could be established statistically by comparing the amounts recovered in bait and TAP-alone control eluates. Proteins which were significantly enriched are depicted in the upper right part of the graph (using a p-value threshold that guarantees an FDR < 0.05). Table 1 and Supplementary Table S5 list all the proteins that were enriched with the baits.

**Table 1.** List of proteins significantly enriched in Mtq2-TAP purifications

GO-terms	WT Mtq2	Mtq2-D77A	Mtq2-N122A
**Bait and its cofactor**	Mtq2, Trm112	Mtq2	Mtq2, Trm112
**90S biogenesis**		Imp4, Snu13	Dhr2, Imp4, Snu13
**60S biogenesis**		Dis3, Nog1, Nog2, Nop2, Nug1, Rix1, Rlp7	Brx1, Dis3, Dbp10, Erb1, Noc3, Nog1, Nog2, Nop2, Npa3, Rlp7, Sqt1, Urb2, Npa2
**40S biogenesis**		Rrp12	Enp1, Rrp12
**Ribosomal subunits export**		Arx1, Nmd3	Arx1, Nmd3
**Large ribosomal subunit proteins**		Rpl30	Rpl5, Rpl9A/B
**Small ribosomal subunit proteins**		Rps1A, Rps6A/B, Rps13,	Rps1A, Rps2, Rps4A/B, Rps6A/B, Rps9A/B, Rps24A/B
**Nuclear pore complex, nuclear transport**		Nup82, Nup84, Nup116, Nup159, Seh1, Yra1	Nup82, Nup84, Nup116, Nup170, Seh1, Gle2
**tRNA modification**		Trm1	Gcd10, Iki3, Rtt10, Trm1
**aa-tRNA ligase**		Gln4, Mes1, Ths1	Grs1, Ths1, Yhr020w
**RNA processing**		Dis3, Mms1, Rtt101	Dis3, Dbp2, Mms1, Rtt101
**mRNA splicing and decay**		Nam7(Upf1), Pat1	Bre1, Ccr4, Dcp2, Edc3, Npl3, Puf3, Pat1
**Translation/regulation**		Gcd1(eIF2BƳ), Gcd6 (eIF2Bϵ), Gcd7 (eIF2Bβ), Rpg1 (eIF3a), Prt1 (eIF3b), Ssd1, Tif4631(eIF4G)	Ecm32, Gcd1(eIF2BƳ), Gcd6 (eIF2Bϵ), Gcd7 (eIF2Bβ), Gcd10, Gcn20, Mkt1-Pbp1, Prt1 (eIF3b), Ssd1, Sup35 (eRF3), Sup45 (eRF1),Tef1 (EF1α), Tif4631(eIF4G)
**Protein folding, modification and degradation**		Rpt1, Rpt4, Ubp3	Bre1, Bre5, Bul2, Cct4, Cct5, Cct6, Cdc48, Gbp1, Nat1, Npl3, Rmt2, Rpt1, Rpt6, Tcp1, Ubp3, Yak1
**TOR pathway**		Iml1, Kog1, Mtc5, Sea4	Iml1, Kog1, Mtc5, Sea4
**Transcription**		Crt10, Dig1, Msi1, Mss116, Pdr1, Puf4, Rpc82, Ssl2, Swi3	Dig1, Hmo1, Mss116, Pdr1, Pog1, Ret1, Rfa1, Rpc40, Rpc82, Ssl2, Ste12
**DNA replication**		Rfc1	Mcm4, Rfc1
**Cell signaling**			Gpb1, Hmo1, Sst2
**Transport**		Mir1, Muk1, Oac1, Sec14, Sla1, Trs130, Ypp1	Ccs1, Fre1, Hxt6/7, Muk1, Pho84, Pil1, Rho3, Rna1, Sfb3, Sla1, Slm1, Trs130, Vma1, Vph1, Ypp1
**Metabolic processes**		Ald5, Gpd2, Ilv2, Mae1, Pho81, Prs1	Ade3, Ald5, Bat2, Bcy1, Cha1, Gas1, Gfa1, Gly1, Ilv2, Ilv3, Lat1, Leu1, Mae1, Mis1, Pdx1, Pho81, Prs1, Rnr1, Snf1, Tps3, Trp5, Urk1
**Cytoskeleton organization**			Act1, Cdc14, Cnm67
**Unknown function**		Ctr86, Ygr250c, Yll032c	Ctr86, Nab6, Pby1, Sdd3, Ygr250c, Yol098c, Yll032c

Among factors involved in early 60S maturation steps, we found Rlp7, Erb1, and Brx1, as well as Nop2 and Noc3. Rlp7, Erb1 and Brx1 act at the same time during nucleolar stages of large subunit assembly (59,76–78). Rlp7 binds to the internal transcribed spacer 2 (ITS2) sequence, and, together with Erb1 and other factors, promotes interactions between the 5.8S rRNA and domain I of 25S, a step necessary for the consecutive 27S pre-rRNA processing (79). Erb1 specifically has a role in the removal of ITS2 and the folding of 25S rRNA domain V, which includes the PTC, and of 5.8S rRNA, important for peptide exit tunnel formation (80). Nop2 and Noc3 bind early in the nucleolus to 25S rRNA domains IV and V, and they also aid in the formation of the PTC (77). In addition, Noc3, together with Noc2, were shown to be important for pre-60S intra-nuclear transport (81). It is unclear why we detected Noc3 but not its known partner Noc2 in our analysis. Among the factors acting at later stages of 60S maturation, we found Nog1, Nog2, Nug1 and Dbp10, which are all involved in PTC and exit tunnel maturation (82,83), and Rix1, which stabilizes ribosomal particles after central protuberance (CP) formation, in a ‘conformational proofreading step’ that precedes nuclear export (84).

In yeast strains expressing TAP-tagged wild-type Mtq2, or Mtq2-N122A, the protein was shown to interact robustly with its known co-activator Trm112 (Figure 3) (25,38), which validated technically our affinity purification scheme. With an FDR of < 5%, this conclusion could not be reached confidently for Mtq2-D77A (although Trm112 appeared just under the cut-off, see graph). The reduced interaction of this Mtq2 mutant with Trm112 is compatible with our former observation that a corresponding mutant of yeast Trm9 (D72A) interacted more weakly with Trm112 (38). As SAM is close to the Mtq2-Trm112 interface, the loss of SAM binding might explain the weaker recovery of Trm112 in Mtq2-D77A purification (Figure 3B).

The eluates from Mtq2-D77A and Mtq2-N122A appeared particularly enriched in factors involved in 60S ribosomal subunit biogenesis (Figure 3B, C). This observation points towards a direct role of Mtq2 in 60S maturation. Trm112 apart, wild-type Mtq2 was not found to interact with any ribosome assembly factors, indicating that the protein interacted only transiently with precursor ribosomes (Figure 3A, Table 1 and Supplementary Table S5). This probably explains why Mtq2 has not been identified in previous pre-60S purifications despite them being rather comprehensive (60).

Overall, factors that interact significantly with Mtq2-D77A and Mtq2-N122A were involved in early, as well as in late steps of 60S maturation, indicating that the protein remained associated with maturing ribosomes for a certain amount of time during ribosomal subunit biogenesis. Many of the interacting factors are known to be important for formation of the functional centers on the large ribosomal subunit, including the peptidyl transferase center (PTC), the peptide exit tunnel (PET), and central protuberance (CP). The CP is a remarkable structural feature of the 60S subunit that is important for transmission of allosteric information between the functional centers of the large subunit and between the small and large subunits. In addition, we found a subunit of the RNA exosome (involved in RNA 3′ end maturation, degradation, and surveillance), factors involved in nucleo-cytoplasmic export of ribosomal subunits, and nucleoporins.

Among the RNA exosome components, we found Dis3 (Rrp44), a catalytic subunit of the complex directly involved in ITS2 processing through formation of the 3′ end of 5.8S (see Supplementary Figure S1, Figure 6B, and below). The RNA exosome has been captured by cryo-EM on maturing 60S precursor subunits (62), and its presence on pre-60S ribosomes purified from *mtq2* mutant cells suggested they were undergoing ITS2 processing and/or were being degraded. The presence of factors that act rather late in the maturation of 60S indicated that pre-60S biogenesis was blocked upon loss of Mtq2 activity. Consequently, Mtq2-containing particles that were enriched in these assembly factors accumulated in the nucleus. This correlated with the presence of two subunit export receptors, namely Nmd3 and Arx1 (63,64), and of several nucleoporins (Nup82, Nup84, Nup116, Nup159, Nup170 and Seh1), in precursor subunits purified from Mtq2-D77A and Mtq2-N122A cells indicated they were late nuclear pre-60S that are inefficiently exported to the cytoplasm. Indeed, all these nucleoporins (excepted Nup84) map a path through the nuclear pore complexes.

In conclusion, by using yeast strains expressing as their sole source of Mtq2 a catalytically-deficient allele of the protein fused to a high affinity purification tag (either the D77A or the N122A mutation), we revealed that Mtq2 was physically associated with maturing 60S subunits. The presence of numerous 60S assembly factors in the eluates indicated that ribosome biogenesis was compromised in the absence of Mtq2-mediated methylation, leading to the accumulation of precursor ribosomes.

Since we found in the eluates assembly factors known to belong to early, intermediate and late 60S precursors, we wanted to deepen our knowledge on the kinetics of association of Mtq2 with pre-ribosomes. For this, we performed reciprocal coprecipitation in Mtq2-N122A cells choosing as bait markers that label early, intermediate, or late 60S nuclear precursors; we used Nop2, Nog2 and Nmd3, respectively (61). Each protein was expressed as a TAP-tag fusion, as described above for Mtq2, and copreciptation assays performed. The eluates were tested by Western-blotting for the presence of Mtq2. The protein was only detectably identified in the Nmd3 eluate, suggesting that it was likely to be more abundant in late nuclear accumulated pre-60S ribosomes than in early or intermediate ones (data not shown).

### The methylation activity of Mtq2 is required for pre-60S nucleolar release and nuclear export

The striking presence of several nucleoporins, and of factors important for pre-60S export from the nucleus to the cytoplasm (Arx1 and Nmd3) (Figure 3B, C and Table 1) suggested that in cells that have loss of Mtq2 function, pre-ribosomal subunits are inefficiently exported and possibly blocked in the nucleus or the nuclear pore complexes.

**Figure 4.**
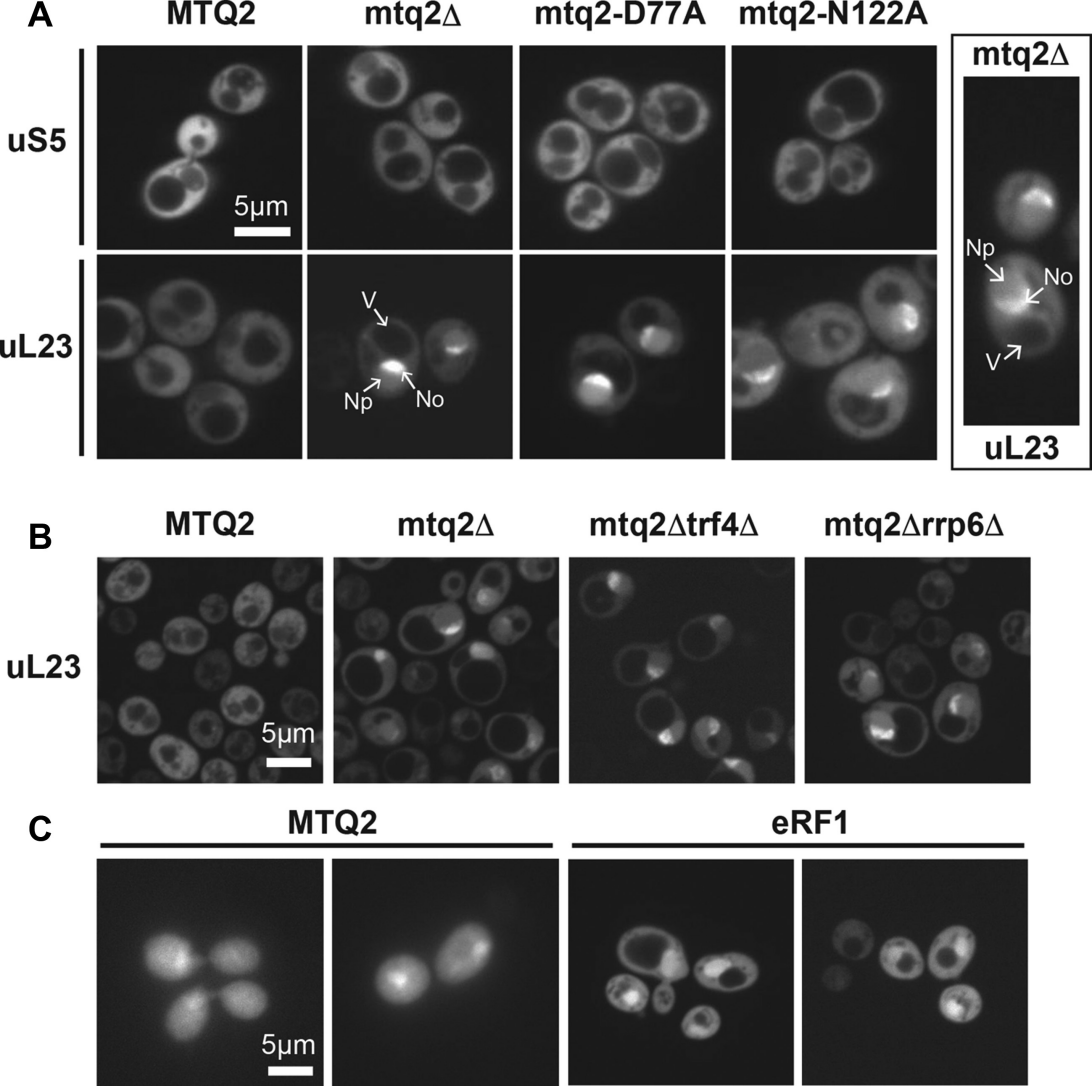
The methylation activity of Mtq2 is required for pre-60S nucleolar release and nuclear export, and Mtq2 and eRF1 can be detected in the nucleus. (**A**) Precursors to large ribosomal subunits accumulate in the nucleolus and nucleus in the absence of functional Mtq2. To monitor ribosomal subunits in cells, the indicated yeast strains were transformed with a construct expressing a green fluorescently-tagged ribosomal protein uS5-GFP or uL23-GFP to follow the small and large ribosomal subunit, respectively. A clear nucleolar and nucleoplasmic accumulation of the uL23 reporter is seen in the absence of Mtq2 function. The nucleolus (No), nucleoplasm (Np), and the vacuole (V) are highlighted. In all panels, images were captured by fluorescence microscopy in spin disc mode at 63×. Scale bar, 5 μm. Inset, 100x. (**B**) Nucleolar surveillance inactivation upon deletion of TRF4 or RRP6 does not increase the load of nucleolar-, or nucleoplasmic-restricted pre-60S observed in cells lacking functional Mtq2. Scale bar, 5 μm. (**C**) Mtq2 and eRF1 can be detected in the nucleus. Mtq2-GFP and eRF1-GFP are localized throughout the cells with nuclear-enrichment. A plasmid expressing an Mtq2-GFP or eRF1-GFP fusion was transformed into wild-type cells. Scale bar, 5 μm.

To test this hypothesis, we monitored the subcellular distribution of ribosomal subunits by transforming the *mtq2* mutants with a plasmid expressing a ribosomal protein fused with the green fluorescent protein (GFP) (65). Small and large ribosomal subunits were monitored using as a proxy uS5-GFP and uL23-GFP, respectively. In wild-type cells, both uS5-GFP and uL23-GFP were largely localized in the cytoplasm where mature ribosomal subunits accumulate (Figure 4A). In cells lacking Mtq2, or expressing the catalytically defective alleles of the protein (D77A or N122A), the cellular distribution of uS5-GFP was not affected as it remained predominantly cytoplasmic (Figure 4A). In contrast, in the *mtq2* mutants, the uL23-GFP construct accumulated in the nucleus displaying a particularly strong nucleolar enrichment, which was visible as a crescent-shaped signal in budding yeast (Figure 4A, inset).

Surveillance mechanisms are active during the entire process of ribosome biogenesis, from the initial nucleolar steps to the final cytoplasmic ones (66). Nuclear-restricted pre-ribosomes are notably known to be degraded by the RNA exosome, with its subunit Rrp6 playing a major role. To test if inactivation of nucleolar surveillance might lead to an increased ‘load’ of nuclear-restricted pre-ribosomes, we inspected the subcellular distribution of pre-60S ribosomes (again using uL23 as proxy) in *mtq2*Δ cells in which we also removed Rrp6. We did not observe any significant differences (Figure 4B). The exosome was recruited by TRAMP complexes, and deletion of its Trf4 subunit in conjunction with Mtq2 also did not reveal any differences (Figure 4B).

Having established Mtq2 as a nuclear pre-60S-associated factor important for their nucleolar release and to a lesser extent its export to the cytoplasm, we were intrigued that eRF1, the only known substrate of Mtq2 thus far, functioned as a release factor during translation in the cytoplasm. It should be noted that the exact role of eRF1 methylation and the exact place where this modification takes place in cells are not known. This prompted us to investigate the subcellular distribution of Mtq2 and eRF1.

Wild-type cells were then transformed with a plasmid expressing a GFP-tagged version of the protein and the fluorescent signal observed by microscopy (Figure 4C). The GFP-tag was fused at the carboxyl terminal end of the protein and the expression driven from the constitutive *ADH1* promoter. Both proteins were detected throughout the cells, including the cytoplasm, and it was also present in the nucleus (Figure 4C). It should be noted that our attempts to localize Mtq2 and eRF1 using GFP constructs expressed directly from the chromosome from the endogenous promoter did not succeed. Constructs expressed from the *ADH1* promoter are mildly overexpressed, so caution must be applied when interpreting the detected signal. For Mtq2, nonetheless, the observed nuclear localization was consistent with its physical presence in nucleolar and nuclear 60S precursor subunits (see Figure 3) and with localization of murine MTQ2 (67).

### The methylation activity of Mtq2 is required for pre-rRNA processing

**Figure 5.**
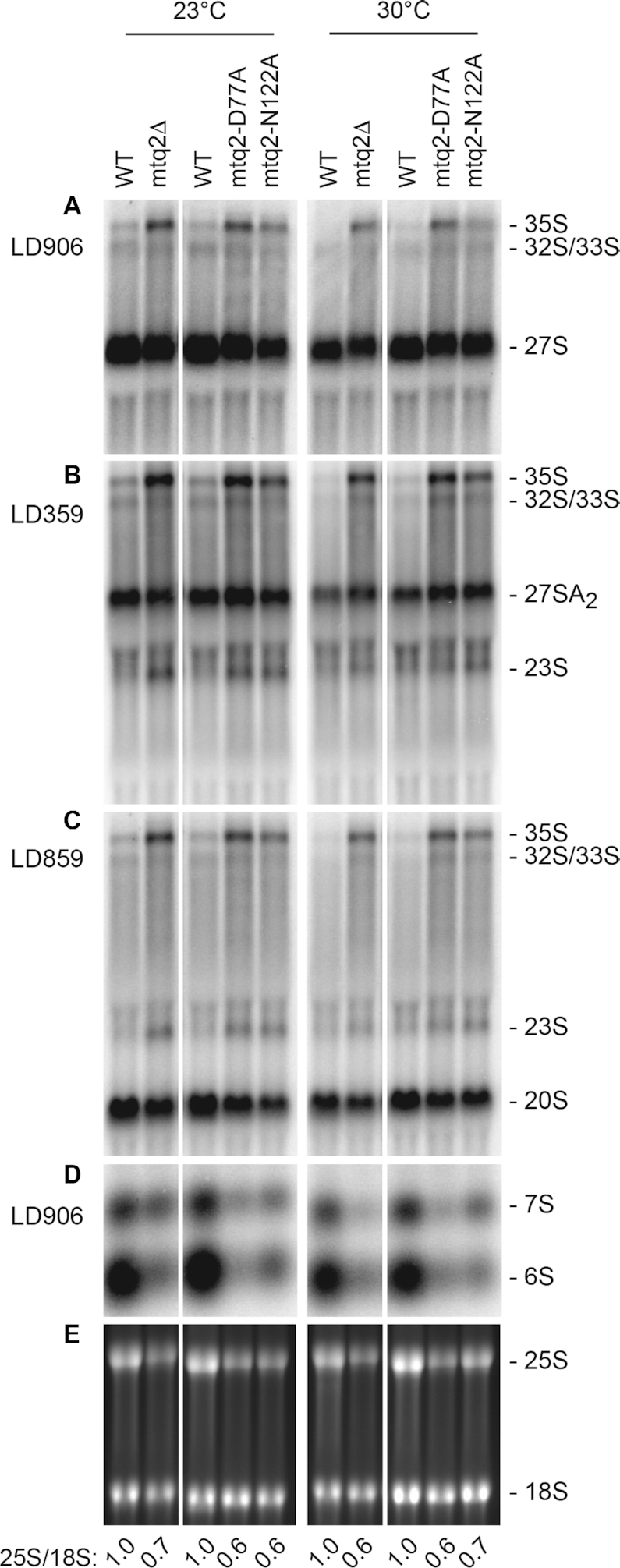
The methylation activity of Mtq2 is required for pre-rRNA processing. Total RNA extracted from the indicated yeast strains grown in glucose-based medium (YPD) at 23 or 30°C were separated on denaturing agarose gels and analyzed by northern blotting with specific probes (A–D), or revealed by ethidium bromide staining (**E**). The probes used are LD906 (panels **A** and **D**), LD359 (panel **B**), LD859 (panel **C**). The RNA species detected are indicated. See Supplementary Figure S1 for details. (E) Ethidium-bromide-stained agarose gel of samples described above, showing the 18S and 25S rRNAs. The 25S/18S ratio was extracted from electropherogramms.

**Figure 6.**
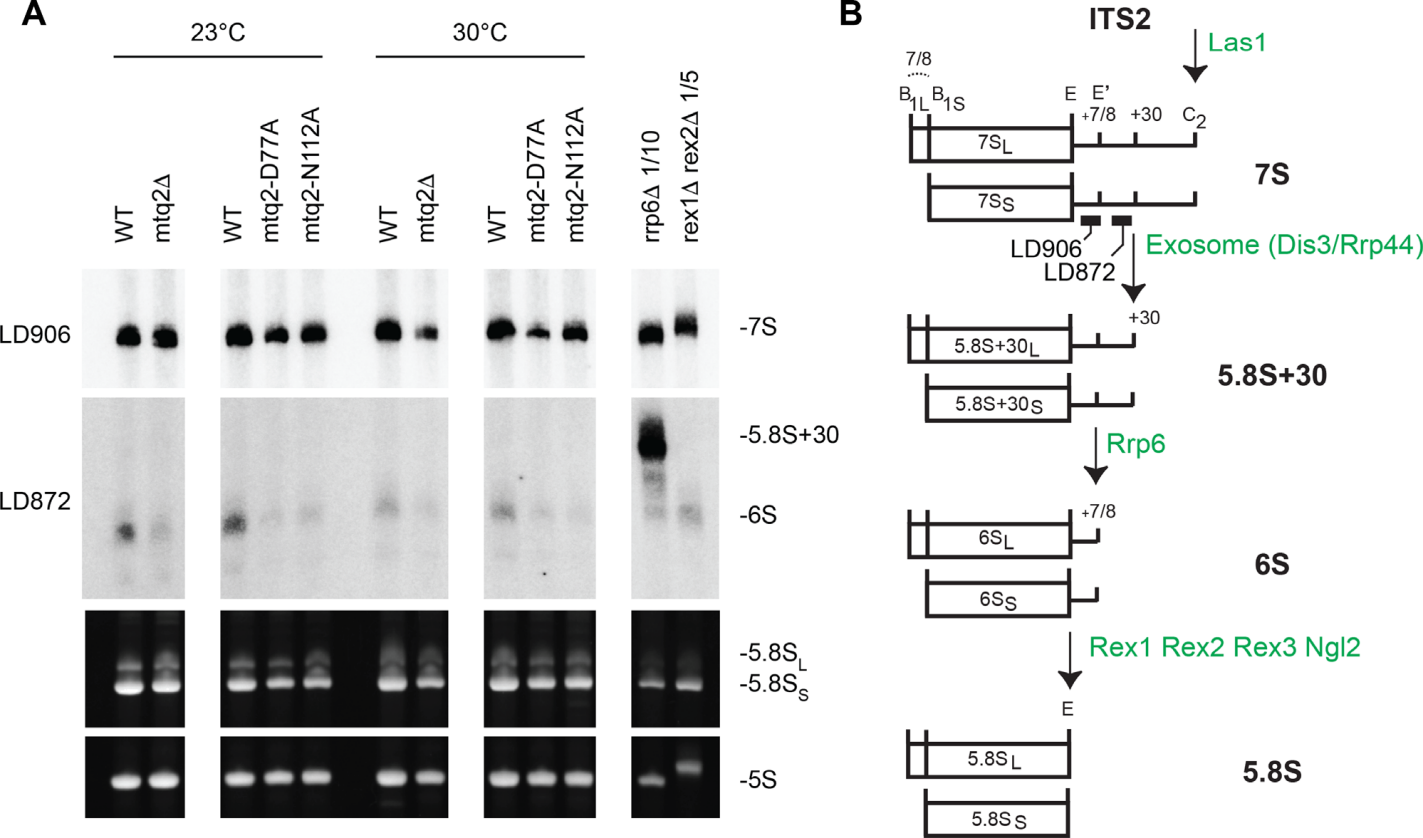
Mtq2 is required for 6S production. (**A**) Total RNA extracted from the indicated yeast strains grown in glucose-based medium (YPD) at 23 or 30°C were separated on denaturing acrylamide gels and analyzed by northern blotting with specific probes (LD906 and LD872, top panels), or revealed by ethidium bromide staining (lower panels). The RNA species detected are indicated. Note that in budding yeast there are two forms of 5.8S (5.8S_S_ and 5.8S_L_) that differ at their 5′ end by the presence of a 7/8-nucleotide-long extension (see B). (**B**) The major steps of internal transcribed spacer 2 (ITS2) maturation. Important intervening factors indicated in green. The position of the probes used in (A) are shown.

In budding yeast, three out of the four mature rRNAs are produced by extensive processing from a single long polycistronic transcript synthesized by RNA polymerase I (Supplementary Figure S1) (68). We performed northern blotting to address a potential role of Mtq2 in pre-rRNA processing. Total RNA extracted from cells lacking Mtq2, or expressing the catalytically-defective alleles D77A or N122A, and from isogenic wild-type control cells, was resolved on denaturing agarose or polyacrylamide gels and analyzed by northern blotting (Figures 5 and 6). To detect all major pre-rRNA processing intermediates, we used a series of radioactively-labelled probes (see Supplementary Figure S1 for a detailed pre-rRNA processing pathway, and probes used). The analysis was conducted both at the physiological temperature of 30°C, and at the lower temperature of 23°C (temperature at which the growth defect was more pronounced, see Figure 1E).

The most striking effect observed in the *mtq2* mutants was a reduction in the steady-state accumulation of the 25S rRNA (Figure 5E) clearly visible on ethidium-bromide-stained agarose gels and confirmed by a reduced 25S/18S ratio. The adverse effects on mature rRNA production were explained by specific processing defects, in particular at sites A_0_, A_1_ and A_2_ (see Supplementary Figure S1). This was illustrated by the systematic accumulation in all *mtq2* mutants analyzed at both temperatures tested of the 35S primary transcript (Figure 5A–C) and by the formation of 23S, which resulted from direct cleavage of the primary transcript at site A_3_ (Figure 5B and C). The 23S extends from the +1 transcription start site to site A_3_ in ITS1 (see Supplementary Figure S1); it is only formed in the absence of cleavage at sites A_0_/A_1_/A_2_. That cleavage at site A_3_ was indeed very efficient in *mtq2* mutants was confirmed by primer extension (Supplementary Figure S2). The 23S RNA is normally not further processed but rather degraded (69). Thus, it forms at the expense of the 20S pre-rRNA and 18S rRNA, and these two species were mildly reduced in *mtq2* mutants. The effects on processing at A_0_–A_2_ are likely indirect since Mtq2 is associated with 60S ribosomal subunit precursors; such feedback inhibitions on early cleavages affecting primarily small ribosomal subunit biogenesis of factors involved in large ribosomal subunit maturation have been observed regularly (68). The accumulation of the 27SA_3_ precursor detected by primer extension indicated that its conversion to 27SB_S_ was slowed in *mtq2* mutants (Supplementary Figure S2), which is in agreement with the notion that *mtq2* mutants are defective for large ribosomal subunit biogenesis.

The involvement of Mtq2 in ribosome biogenesis was further analyzed by metabolic labeling (Supplementary Figure S3). The pulse-chase performed at 23°C in *mtq2*Δ cells revealed a severely reduced incorporation of radiolabelled nucleotides, in agreement with the reduced cell growth (Figure 1), and a delay in the production of 18S and 25 rRNAs (Supplementary Figure S3, 18S and 25 rRNAs appear after 10 min of chase in the isogenic control while they are only formed at the 20 min time point in the mutant).

The three *mtq2* mutant strains were also defective for late maturation of internal transcribed spacer 2 (ITS2) that separates the 5.8S and 25S rRNAs on pre-rRNA intermediates (Figure 6 and Supplementary Figure S1). Maturation of ITS2, involves dozens of *trans*-acting factors, including endo- and exoRNases acting sequentially (70). ITS2 maturation is initiated on the 27S pre-rRNA by endonucleolytic cleavage at site C_2_ (51,71), performed by Las1, generating the 7S and 26S intermediates (see Supplementary Figure S1). The 7S is progressively trimmed at its 3′ end into mature 5.8S rRNA by the RNA exosome and other factors. In wild-type cells, the maturation of 7S into 5.8S involves the accumulation of discrete metastable species, including the 6S, which corresponds to premature forms of 5.8S rRNA extended on 3′ end by 7/8 nucleotides (Figure 6B) (51). We observed a systematic reduction in all *mtq2* mutants analyzed at both temperatures tested in the steady-state levels of 7S and 6S (Figure 5D and Figure 6A). The effect was particularly strong for 6S and was accompanied by a mild reduction of 5.8S (Figure 6A). As control, total RNA extracted from *rrp6Δ* and *rex1Δrex2Δ* cells were used to visualize the 5.8S+30 and 6S, respectively. Because the 27S pre-rRNAs accumulated to near normal levels in *mtq2* mutants (Figure 5A), we concluded that the initial cleavage at site C_2_ in ITS2 was largely unaffected, but that once it occurred the maturation of 60S subunit was blocked. We observed that inactivating the exosome (*rrp6Δ*) in cells lacking Mtq2 largely restored the levels of 6S, which was compatible with the idea that blocked pre-60S are subjected to increased turnover (Supplementary Figure S4).

## DISCUSSION

### The Mtq2-Trm112 methyltransferase is a novel ribosome assembly factor required for 60S subunit biogenesis

When we initiated this work, the Mtq2–Trm112 methyltransferase was only known to modify the translation termination factor eRF1. It was also known that loss of Mtq2 leads to cell growth defects that could not be explained alone by a putative role in translation since eRF1 methylation (but not the residue that is modified, see below) seems dispensable to this process, at least in yeast (5,6). We therefore investigated other possible explanations for the severe growth defect associated with the deletion of *mtq2*, and we found ribosome biogenesis to be severely affected. Specifically, we report that Mtq2 is a novel ribosome assembly factor required for large ribosomal subunit biogenesis. We demonstrate that catalytically-dead Mtq2 mutant (D77A) phenocopies the *mtq2* deletion, which incriminates the methylation function in the phenotypes. We show that Mtq2 is associated with nuclear 60S precursor subunits, and that its catalytic activity is required for efficient production of 5.8S and 25S rRNAs. In the absence of the catalytic function of Mtq2, 60S precursor subunits fail to be released from the nucleolus and to be exported to the cytoplasm. Instead, we suggest they are degraded, at least partly.

Our findings also rationalize a former observation, including from our groups, showing that Trm112 is involved in the biogenesis of both the small and large ribosomal subunits (27,29). At that time, only the small subunit maturation defects could be explained and invoked the loss of metabolic stability of the associated 18S rRNA m^7^G methyltransferase Bud23 upon deletion of the *TRM112* gene. Indeed, Bud23 gains metabolic stability through formation of a complex with Trm112, which involves formation of a β-zipper between main chain atoms (14). For the large ribosomal subunit biogenesis defect, we had no explanation to offer at the time (14). Considering that, as for Bud23, the levels of Mtq2 are also strongly reduced in *trm112*Δ cells (29), our novel findings that Mtq2 is part of nuclear pre-60S subunits being required for the efficient production of 5.8S and 25S rRNAs (Figures 5 and 6) explain molecularly why Trm112 is also required for large ribosomal subunit production.

### Mtq2 is associated with nuclear 60S subunit precursors

Upon defining the interactome of Mtq2, we realized that the protein is associated with nuclear 60S precursor subunits (Figure 3). This observation was essential in establishing a direct involvement of Mtq2 in ribosome biogenesis. In particular, affinity purifications performed with catalytically inactive forms of Mtq2 led to the identification of numerous 60S assembly factors (Figure 3B and C, Table 1). These factors are not detected when the catalytically active form of Mtq2 is used as a bait (Figure 3A), indicating that the nature of the interactions is presumably transient in unperturbed conditions. We therefore suggest that 60S precursor subunit assembly is blocked in the absence of Mtq2 methylation activity and that unfaithfully assembled subunits, which are not efficiently released from the nucleolus and not exported to the cytoplasm, are degraded. The large subunit precursor export defect is consistent with the presence on purified Mtq2 particles of the transport receptors Nmd3 and Arx1, and of several nucleoporins. The conspicuous presence in the Mtq2 mutant eluates of Mms1 and Rtt101, which are both involved in 25S non-functional ribosomal decay (72), and of the Dis3 exosome catalytic subunit, involved in nucleolar surveillance, is also interesting as it suggests subunits accumulated in *mtq2* mutants may be discarded using these pathways.

Finally, it is interesting that Mtq2 is associated with early and late-assembly factors involved in the maturation of functional centers on the large ribosomal subunit: the peptidyl transferase center (Nog1, Nog2, Nug1 and Dbp10), the peptide exit tunnel (Nog1), and the central protuberance (Rix1). This suggests that Mtq2 may play a role in surveilling the formation of these important ribosomal sites (see below).

### Models for Mtq2 intervention during late nuclear 60S subunit maturation

Currently, the only characterized substrate of Mtq2 in yeast is the translation termination factor eRF1 (1,6,25), which was co-enriched in our affinity purifications together with eRF3 using catalytically inactive Mtq2N122A as a bait (Table 1). Because eRF1, together with eRF3-GTP and Rli1/ABCE1, is acting during translation in the cytoplasm, it was assumed that this is where methylation takes place; however, this was never demonstrated. On the basis of our observations that: (i) Mtq2 and eRF1 can be detected in the nucleus (Figure 4C); (ii) Mtq2 is part of nuclear pre-60S particles (Figure 3) and (iii) the catalytic activity of Mtq2 is required for efficient 60S production (Figures 5 and 6); we propose two alternative models for the involvement of Mtq2 in 60S subunit biogenesis.

In the first model, we suggest Mtq2 might methylate eRF1 in the nucleus where it might play a quality control role to assess formation of the peptidyl transferase center. This role may involve ‘probing’ of the binding pocket where it will subsequently bind on mature ribosomes during translation termination. eRF1 methylation is not essential, so such a ‘quality control’ role would only optimize the process rather than being strictly required for it. Elongation factor like 1 (EFL1), which is also non-essential and whose deletion also confers slow growth (73), has been proposed to play a similar role for proof-reading of the binding sites of translational elongation factors once 60S precursor ribosomes reach the cytoplasm (73). In an alternative model, we propose Mtq2 might methylate an unknown yet to be identified substrate involved in 60S biogenesis in the nucleus. Such a novel substrate might be a ribosome assembly factor or a ribosomal protein. Future work will help us understand which of the two proposed models is the correct one. So far, we could not test directly our first hypothesis as it was not possible to uncouple the methylation of eRF1 from its role in translation termination (the eRF1 variants produced were either lethal for cell growth, or defective for translation termination, VH, unpublished).

As shown for human HEMK2 (74,75) and yeast Hpm1 (8), yeast Mtq2 may have additional substrates that remain to be identified. In considering the nature of the active site of Mtq2, which is highly negatively charged, and the structure and composition of the eRF1 GGQ motif, we believe it is reasonable to propose the existence of putative novel Mtq2’s substrates carrying an exposed Gln residue within a positively charged electrostatic environment (38). Directly relevant to this assumption is the characterization of a modification consensus sequence in an *in vitro* study based on the methylation of a peptide library by murine MTQ2. The identified consensus is GQX_3_R with: (i) a preferred Ser, Arg, or Gly at position +1 (with respect to the modified Gln); (ii) a preferred Arg at +7 and (iii) the strict absence of a Pro within the five consecutive amino acids following the Gln (74). Among the novel protein substrates of metazoan MTQ2, RRP1 is of potential interest due to its known involvement in 60S biogenesis. Unfortunately, the region containing the methylated glutamine on human RRP1 is not conserved in the homologous yeast protein.

Upon scanning the proteins interacting with Mtq2 mutants with the methylation consensus established in mouse, we identified several candidate proteins (Table 2). While none of them entirely fulfills the mouse consensus, four have drawn our attention: Nug1 (containing a GGQ flanked by Arg and Lys), Nup116 and Rix1 (GGQ motif in a context different from the consensus) and Ygr250c (GQX3R motif). We are currently investigating whether any of these proteins might be a novel substrate of Mtq2. However, one cannot rule out that yeast Mtq2 might also modify a lysine side chain as recently demonstrated for its human orthologue HEMK2, which methylates lysine 12 on histone 4 (75).

**Table 2.** List of proteins identified in Mtq2-TAP purifications containing a GGQ or a GQX_3_R motif

Protein	Sequence around Gln	GGQ	Basic environment	GQ(X)_3_R motif
**Sup45 (eRF1)**	DLPKKHGRGGQSALRFARLREEKR	+	+++	+
**Nug1**	INALLARRGGQSKACPVGNEAGVT	+	++	−
**Nup116**	FGSTGSTFGGQQQQQQPVANTSAF	+	−	−
**Rix1**	NPLVLSTHGGQLLAAIYSRLEQKT	+	−	−
**Bul2**	SFEMYNALHRHIPQG NVDPDRHD	−	−	+
	LTINSTRSGGQSLHSSSDTNISQI	+	−	−
**Cct5**	DLPAVRWVGGQE LEHIAISTNGRI	+	−	−
**Gcn20**	LQKMQLLSGGQKSRVA FAALCLNN	+	+	−
**Ilv2**	DTSFVGLTGGQIFNEMMSRQNVDT	+	−	−
**Mes1**	IDEWRAKYGGQQV	+	+	−
**Puf4**	SGAAVATQGGQNLNPLINDNSMKV	+	−	−
**Rnr1**	RSYLLRINGQVAERPQHLIMRVA	−	−	+
**Ubp3**	PSPISKLFGGQFRSVLDIPNNKES	+	+	−
**YGR250C**	RESFSEGRGQRVPRFMGNKFDMY	−	+	+

The table highlights the presence of a GGQ or a GQX_3_R motif (74), and the presence of a positively charged environment, which is known to be favorable to methylation, in putative Mtq2 substrates.

In summary, we have identified the Mtq2–Trm112 complex as a novel ribosome assembly factor required for large ribosomal subunit biogenesis. We have demonstrated that Mtq2 is associated with maturing nuclear 60S precursor subunits, and that its enzymatic activity is required for pre-60S maturation, nucleolar release, and export to the cytoplasm. We speculate that in the absence of Mtq2 activity, the 60S maturation pathway is blocked, leading to the nuclear accumulation and degradation of unfaithfully assembled subunits. Whether Mtq2 fulfills its function in ribosome biogenesis by methylating eRF1 as part of a quality control mechanism aimed at proof-testing the eRF1 binding site on maturing subunits, or by methylating another yet to be identified substrate remains to be established.

## DATA AVAILABILITY

Complete proteomic datasets have been deposited to the ProteomeXchange Consortium database (46) and can be accessed at PXD018733.

## Supplementary Material

gkaa972_Supplemental_File
