# Supplementary material for: The catalytic activity of the translation termination factor methyltransferase Mtq2-Trm112 complex is required for large ribosomal subunit biogenesis
Source: Nucleic Acids Res. 2020 Nov 9;48(21):12310–25. doi: 10.1093/nar/gkaa972 (PMC7708063; doi:10.1093/nar/gkaa972)
Supplement: gkaa972_Supplemental_File [file gkaa972_supplemental_file.pdf]

SUPPLEMENTARY FILES

The catalytic activity of the translation termination factor methyltransferase Mtq2-Trm112 complex is required for large ribosomal subunit biogenesis

Caroline Lacoux[1,*], Ludivine Wacheul[2,*], Kritika Saraf[2], Nicolas Pythoud[3], Emmeline Huvelle[1], Sabine Figaro[1], Marc Graille[4], Christine Carapito[3], Denis L.J. Lafontaine[2,#], Valérie Heurgué-Hamard[1,#]

[1]UMR8261 (CNRS, Université de Paris), Institut de Biologie Physico-Chimique, 13 rue Pierre et Marie Curie, 75005 Paris, France

[2]RNA Molecular Biology, ULB Cancer Research Center (U-CRC), Center for Microscopy and Molecular Imaging (CMMI), Fonds de la Recherche Scientifique (F.R.S.-FNRS), Université Libre de Bruxelles (ULB), Belgium

[3]Laboratoire de Spectrométrie de Masse Bio-Organique (LSMBO), UMR 7178, IPHC, Université de Strasbourg, CNRS, Strasbourg, France

[4]Laboratoire de Biologie Structurale de la Cellule, CNRS, Ecole polytechnique, IP Paris, Palaiseau, France

*The first two authors contributed equally to this work

#The last two authors contributed equally to this work

Correspondence:

denis.lafontaine@ulb.ac.be

heurgue@ibpc.fr

SUPPLEMENTARY METHODS

Proteomic sample preparation

After denaturation at 100 °C for 5 min in loading buffer (2% SDS, 0.1 M DTT, 10% glycerol, 62.5 mM Tris-HCl pH 6.8), all the protein extracts of each sample was concentrated in one band on a 4% SDS-PAGE gel. The gels were fixed with 45% methanol/3% acetic acid and stained with colloidal Silver Blue. Each band was excised and cut into four pieces prior to *in-gel* digestion. The gel pieces were washed four times with 100 µl of 75% acetonitrile and 25% NH_4HCO_3 at 25 mM and dehydrated with 50 µl of acetonitrile. The cysteine residues were reduced by adding 10 mM DTT for 30 min at 60°C and 30 min at room temperature, and alkylated by adding 55 mM iodoacetamide for 20 min in the dark. The bands were then washed three times by adding 50 µl of 25 mM NH_4HCO_3 and 50 µl of ACN. After two dehydrations with 50 µl of ACN, gel pieces were stored at -20 °C prior to enzymatic digestion. Proteins were cleaved in an adequate volume to cover all the gel pieces with 25 µl of a modified porcine trypsin solution (Promega) at 0.004 µg/µl. Digestion was performed overnight at 37°C. Tryptic peptides were extracted twice under agitation, first with 40 µl of 60% ACN in 0.1% formic acid (FA) for 1 h and then with 30 µl of 100% ACN for 1 h. The collected extracts were pooled, the excess ACN was vacuum dried, and the samples were resolubilized with 8 µl of H_2O/ACN/FA (98/2/0.1 v/v/v).

NanoLC-MS/MS Analysis

NanoLC-MS/MS analyses were performed on a nanoAcquity UPLC device (Waters Corporation, Milford, USA) coupled to a Q-Exactive Plus mass spectrometer (Thermo Scientific, Bremen, Germany). Peptide separation was performed on an ACQUITY UPLC BEH130 C18 column (250 mm × 75 µm with 1.7 µm diameter particles) and a Symmetry C18 pre-column (20 mm × 180 µm with 5 µm diameter particles, Waters). The solvent system consisted of 0.1% FA in water (solvent A) and 0.1% FA in ACN (solvent B). The samples were loaded into the enrichment column over 3 min at 5 µL/min with 99% of solvent A and 1% of solvent B. Peptides were eluted at 450 µl/min with the following gradient of solvent B: from 1 to 8% over 2 min, 8 to 35% over 77 min, and 35 to 90% over 1 min. The MS capillary voltage was set to 1.8 kV at 250 °C. The system was operated in Data-Dependant Acquisition (DDA) mode with automatic switching between MS (50 ms/scan over a 300–1800 m/z range with R = 70,000) and MS/MS (100 ms/scan over a 200–2000 m/z range with R = 17,500) modes. The ten most abundant ions (intensity threshold 2×10^5) were selected on each MS spectrum for further isolation and higher energy collision dissociation (HCD) fragmentation, excluding unassigned and monocharged ions. The dynamic exclusion time was set to 60 s.

Quantitative Proteomic Data Analysis

Raw data collected during nanoLC-MS/MS were processed using MaxQuant (version 1.6.0.16) (1). Peaks were assigned with the Andromeda search engine with trypsin/P specificity. The database used for the searches was extracted from UniProtKB-SwissProt database and included all *Saccharomyces cerevisiae* entries (9 April 2018; Taxonomy ID=4932; 7,905 entries). The minimum peptide length required was seven

amino acids and a maximum of one missed cleavage was allowed. The precursor mass tolerance was set to 20 ppm for the first search and 4.5 ppm for the main search. The fragment ion mass tolerance was set to 20 ppm. Methionine oxidation, carbamidomethylation and propionamide of cysteines were set as variable modifications. The maximum false discovery rate was 1% for peptides and proteins with the use of a decoy strategy. The “match between runs” option was used. Peptides with modified methionines, as well as their unmodified counterparts, were excluded from protein quantification. The final protein list was obtained after suppression of contaminants, reverse entries, and proteins only identified with modified peptides. We used the “proteingroups.txt” file with LFQ intensities (normalized intensities). The dataset has been deposited to the ProteomeXchange Consortium via the PRIDE partner repository with the dataset identifier PXD018733 (2).

SUPPLEMENTARY FIGURES

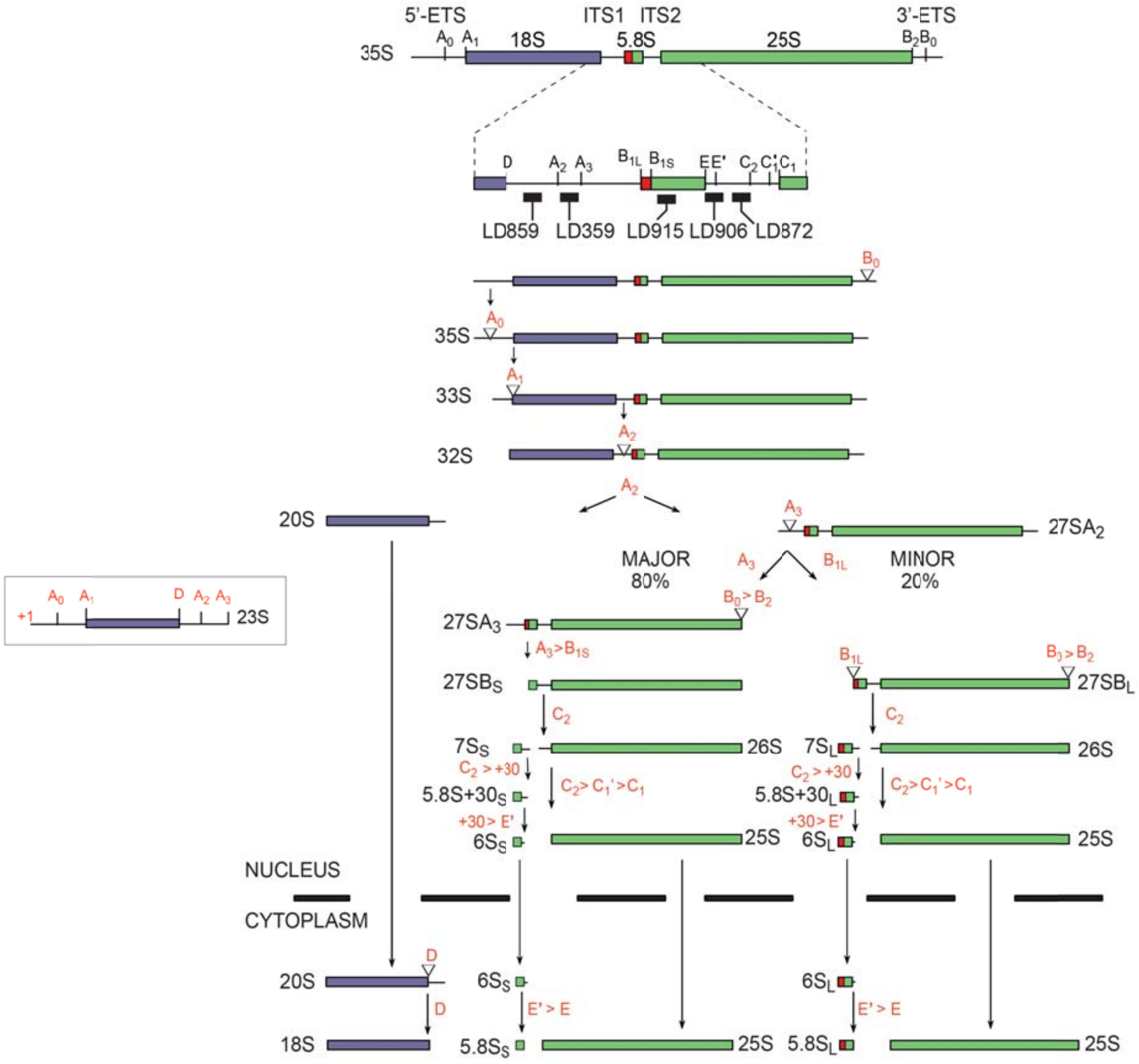

Figure S1: Schematic representation of the yeast pre-rRNA processing pathway

Three out of four mature rRNAs, the 18S (the only RNA component of the small subunit, 40S), the 5.8S and 25S (both belonging to the large subunit, 60S) are produced from a long polycistronic transcript (35S) by RNA polymerase I (Pol I). The fourth rRNA (5S, also part of the large subunit) is produced independently by RNA Pol III (not represented). Within the 35S pre-rRNA, the 18S, 5.8S, and 25S rRNAs are separated by non-coding intervening sequences: the 5' and 3' external transcribed spacers (ETS), and the internal transcribed spacers (ITS) 1 and 2. The ETS and ITS sequences are removed, and the mature ends of the rRNAs produced by extensive processing. The major processing pathways are depicted (see ref. (3) for details). The processing sites (A_0, A_1, etc.), and the probes used in this work (LD859, LD359, LD915, LD906, and LD872) are indicated. There are two forms of 5.8S rRNA, which differ by a 7/8-nucleotides long extension at the 5' of the RNA (represented as a red square). Insert, the aberrant 23S RNA results from premature cleavage at site A_3 in ITS1 in the absence of cleavage at sites A_0, A_1, and A_2.

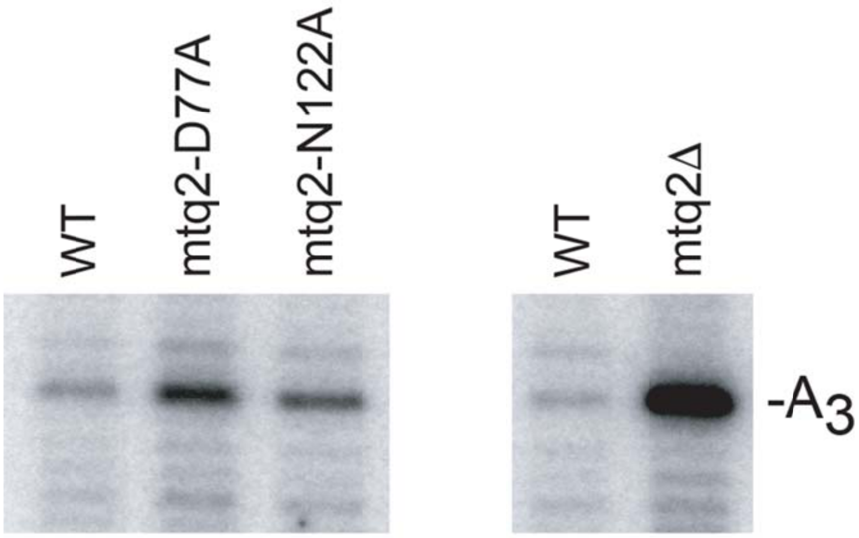

Figure S2: Primer extension analysis of rRNA intermediates ending at processing site A_3

Total RNA extracted from the indicated yeast strains grown in glucose-based medium (YPD) at 30°C was processed in a primer extension assay with oligonucleotide LD915 to reveal processing site A_3 (see Figure S1).

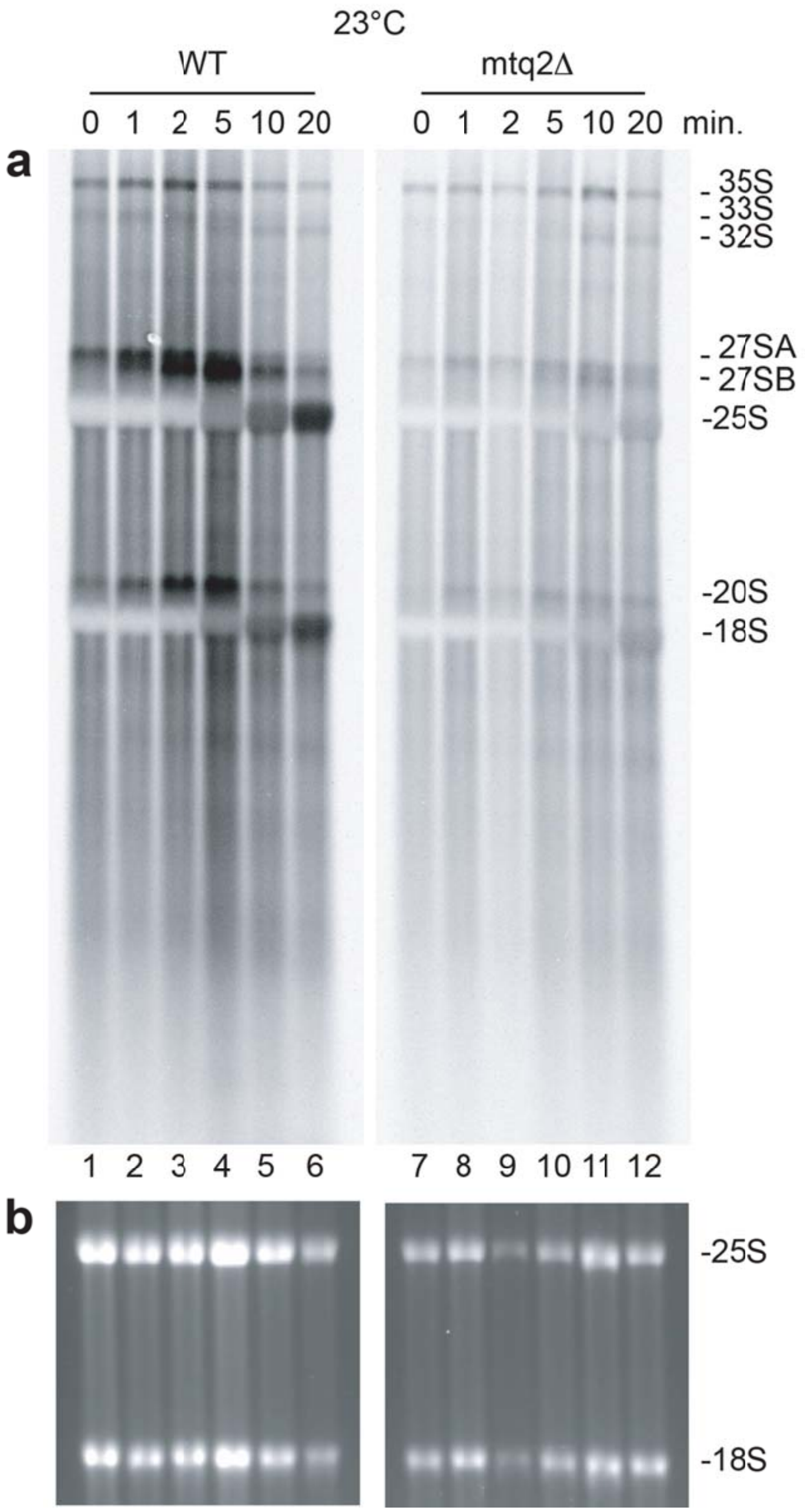

Figure S3: Pulse chase analysis of ribosome biogenesis defect in *mtq2Δ* cells

*mtq2*Δ and isogenic control cells were grown in minimal medium lacking uracil, pulsed with tritiated uracil for 1 min, and then chased with an excess of cold uracil. Total RNA was extracted from cells at 0,1, 2, 5, 10 and 20 min after the chase, separated on a denaturing agarose gel, transferred to a Genescreen membrane and exposed to an imaging plate.

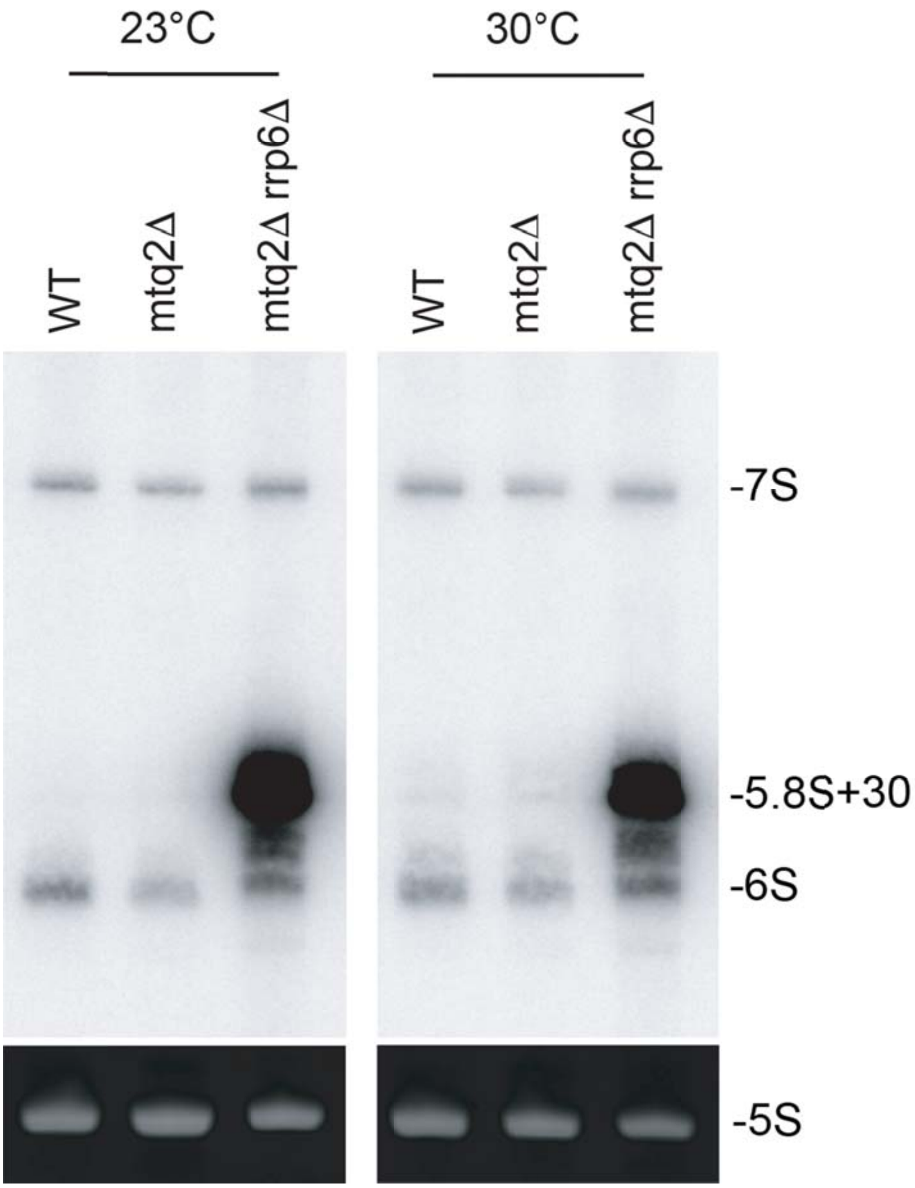

Figure S4: Steady-state level analysis of 6S pre-rRNA in cells lacking Mtq2 in the absence of nucleolar surveillance (*rrp6*Δ)

Total RNA extracted from the indicated yeast strains grown in glucose-based medium (YPD) at 23°C or 30°C were separated on denaturing acrylamide gels and analyzed by northern blotting with probe LD906 or revealed by ethidium bromide staining (lower panel, 5S rRNA loading control). The RNA species detected are indicated.

SUPPLEMENTARY TABLES

Table S1. Oligonucleotides used for the construction of yeast strains and plasmids

Oligonucleotide	Sequence	Strain/ plasmid
Mtq2F3up	GGGATGGGAAGTCCTCAGTGTGTACAGCTTTACAAGGTGAggcg cgccacttctaaa	yVH192<br>yVH440
Mtq2R1low	ACACAGGTTATCAATTATAACGTGAAAGGTTTTGCAACTGgaattc gagctcgtttaaac	
Mtq2-D77A	ATAATACCGCAGGAAAATTCCATCCACTTAGCTGTTG**C**TATCAA CCCATGGGCGCTCG	yVH200
Mtq2-N122A	ATTCGGAATAATCAGGTTGATGTCCTAATATTT**GC**CCCACCATA **C**GTGCCAGCAGAATGT	yVH201
Up tap Mtq2	GAAACGAGAAAAGCGGGATGGGAAGTCCTCAGTGTGTACAGC TTTACAAGGtccatggaaaagagaag	CL3<br>CL5
Down tap Mtq2	CTTTGAGTAAAGACACAGGTTATCAATTATAACGTGAAAGGTTT TGCAACTGtacgactcactataggg	
Mtq2kan571	CTGGTCGTTAGTAAGGTCTC	yVH466<br>yVH468<br>yVH471
Mtq2kan2977	CTCAACATGGAAGGCTTCAC	
Trf4up622	CTCAGAAGTCAGCCAACAAC	yVH443
Trf4down3269C	AACGGGCTTGCCCTTAAATC	

Rrp6up541<br><br>Rrp6down3682C	CACTTCGAGATGAGCTTGAG<br><br>CCACTGGCCTAAACGGATAC	yVH450
Mtq2BamHI-GFPpRS5'<br><br>Mtq2XhoI-GFPpRS3'	TAATATGGATCCACTTAACACAGGGTGAGAAAG<br><br>TTTGCAACTCGAGCCTTGTAAAGCTGTACACACTG	pVH511
eRF1XbaI-GFPpRS5'<br><br>eRF1XhoI-GFPpRS3'	GGAAATACTTCTAGAATGGATAACGAGGTTGAAA<br><br>TTTACTCGAGAATGAAATCATAGTCGGATC	pVH512

Nucleotide sequences from *S. cerevisiae* genome and from plasmid are indicated in capital and small letters, respectively. Nucleotides corresponding to introduced mutations are in bold.

Table S2. Yeast strains used in this study

**Strain**	**Genotype**	**Source**
BY4741	*MAT a his3Δ1 leu2Δ0 met15Δ0 ura3Δ0*	Euroscarf
CL3	*MAT a his3Δ1 leu2Δ0 met15Δ0 mtq2(D77A)::TAP-URA3*	This study
CL5	*MAT a his3Δ1 leu2Δ0 met15Δ0 mtq2(N122A)::TAP-URA3*	This study
FF12	*MAT a his3Δ1 leu2Δ0 met15Δ0 ura3Δ0 mtq2Δ::his3*	(4)
FF19	*his3Δ1 leu2Δ0 ura3Δ0 trm112Δ::kanMX4*	(5)
yVH192	*MAT a his3Δ1 leu2Δ0 met15Δ0 ura3Δ0 mtq2-his3MX6*	This study
yVH200	*MAT a his3Δ1 leu2Δ0 met15Δ0 ura3Δ0 mtq2(D77A)-his3MX6*	This study

yVH201	*MAT a his3Δ1 leu2Δ0 met15Δ0 ura3Δ0 mtq2(N122A)-his3MX6*	This study
yVH440	*MAT a his3Δ1 leu2Δ0 met15Δ0 ura3Δ0 mtq2(N122A)-kanMX6*	This study
yVH443	*MAT a his3Δ1 leu2Δ0 met15Δ0; ura3Δ0; mtq2::kanMX4, trf4::CgHis3*	This study
yVH450	*MAT a his3Δ1 leu2Δ0 met15Δ0; ura3Δ0; mtq2::kanMX4, rrp6::natMX6*	This study
yVH466	*MAT a (his3Δ1) leu2Δ0 met15Δ0 ura3Δ0 NOP2::TAP-HIS3MX6, mtq2(N122A)-kanMX6*	This study
yVH468	*MAT a (his3Δ1) leu2Δ0 met15Δ0 ura3Δ0 NMD3::TAP-HIS3MX6, mtq2(N122A)-kanMX6*	This study
yVH471	*MAT a (his3Δ1) leu2Δ0 met15Δ0 ura3Δ0 NOG2::TAP-HIS3MX6, mtq2(N122A)-kanMX6*	This study
Y04074	*MAT a his3Δ1 leu2Δ0 met15Δ0; ura3Δ0; mtq2::kanMX4*	Euroscarf
YDL2488	*MAT a his3Δ1 leu2Δ0 met15Δ0; ura3Δ0; trf4::CgHis3*	Denis Lafontaine
YDL1833	*MAT a his3Δ1 leu2Δ0 met15Δ0; ura3::CMV-tTA, rrp6Δ::natMX6*	(6)
YDL2618 (TAP-alone)	*MAT a his3Δ1 leu2Δ0 met15Δ0 PrBAT2-TAP*	(7)
YSC1178-202230426	*MAT a (his3Δ1) leu2Δ0 met15Δ0 ura3Δ0 MTQ2-TAP-HIS3MX6*	Open Biosystem
YSC1178-7502608	*MAT a (his3Δ1) leu2Δ0 met15Δ0 ura3Δ0 TRM112-TAP-HIS3MX6*	Open Biosystem
YSC1178-202232980	*MAT a (his3Δ1) leu2Δ0 met15Δ0 ura3Δ0 NOP2-TAP-HIS3MX6*	Open Biosystem
YSC1178-202233224	*MAT a (his3Δ1) leu2Δ0 met15Δ0 ura3Δ0 NOG2-TAP-HIS3MX6*	Open Biosystem
YSC1178-202231551	*MAT a (his3Δ1) leu2Δ0 met15Δ0 ura3Δ0 NMD3-TAP-HIS3MX6*	Open Biosystem

Table S3. Oligonucleotides used for Northern blot and primer extension

Oligonucleotide	Sequence
LD906	TGAGAAGGAAATGACGCT
LD359	TTGTTACCTCTGGGCCC
LD859	CGGTTTTAATTGTCCTA
LD872	GGCCAGCAATTTCAAGTTA
LD915	GCGTTCTTGATCGATGC

Table S4. Doubling times of *mtq2* mutant strains

strain	23°C DT (min)	s.d.	30°C DT (min)	s.d.	37°C DT (min)	s.d.
**BY4741**	167	26	92	3	100	2
**yVH192**	169	23	94	3	102	5
**Y04074 (mtq2Δ)**	319	14	173	9	148	14
**yVH200 mtq2(D77A)**	313	21	163	4	141	9
**yVH201 mtq2 (N122A)**	270	21	118	3	99	14

Doubling times are the average of three or four independent measurements. Strains were grown in YPDA rich medium at indicated temperatures and OD_{600} was measured. DT, doubling time; s.d., standard deviation.

Table S5. Proteins enriched in affinity purification experiments

MTQ2 versus TAP-alone					
**Gene names**	**Accession numbers**	**Peptides**	**Unique Peptides**	**$-\log_{10}$ (p-value)**	**$-\log_2$ (fold-change)**
**Enriched proteins**					
Trm112	P53738	7	7	2,33	4,28
Mtq2	Q03920	20	20	5,21	5,60

mtq2-D77 versus TAP-alone					
**Gene names**	**Accession numbers**	**Peptides**	**Unique Peptides**	**-log$_{10}$ (p-value)**	**-log$_2$ (fold-change)**
**Enriched proteins**					
Sla1	P32790;A6ZKU1	14	14	2,55	2,80
Prt1	P06103;A6ZPJ1	21	21	1,89	2,63
Ald5	P40047;A6ZR27;P32872	24	23	1,81	1,41
Rix1	P38883;A6ZTA3	5	5	2,24	1,95
Ypp1	P46951;A6ZUK8	7	7	2,35	2,11
Rpc82	P32349;A6ZX64	28	28	1,99	1,26
Rps1A	P33442;C7GSL4;B5VNY0 B3RHV0;A7A1W1	21	4	2,47	1,66
Mes1	P00958	14	14	2,43	0,67
Ths1	P04801	15	15	1,95	1,64
Rps13	P05756	6	6	2,46	0,45
Ilv2	P07342	32	32	2,23	2,19
Gcd1	P09032	13	13	2,46	3,01
Rps6B Rps6A	P0CX38;P0CX37	10	10	2,11	1,07
Pdr1	P12383	27	27	2,89	2,41
Gln4	P13188	9	9	2,94	1,36
Rpl30	P14120	5	5	3,16	0,61
Mss116	P15424	26	26	4,58	2,67
Trm1	P15565	10	10	1,82	1,65
Pho81	P17442	16	16	2,13	2,50
Mir1	P23641	7	7	2,15	2,48
Ssd1	P24276	28	28	2,41	2,00
Sec14	P24280	6	6	1,98	2,16
Puf4	P25339	16	16	2,30	3,53
Ctr86	P25355	8	8	2,03	1,52
Pat1	P25644	10	10	3,56	2,45
Nam7	P30771	45	45	1,86	2,47
Oac1	P32332	5	5	2,12	2,13
Gcd6	P32501	27	27	1,93	2,61
Gcd7	P32502	9	9	2,46	2,37
Swi3	P32591	8	8	2,32	1,79
Prs1	P32895	11	11	3,10	1,31
Rpt1	P33299	16	16	1,87	0,63
Mae1	P36013	14	14	3,09	1,93
Sea4	P38164	27	27	2,60	2,17
Rpg1	P38249	23	23	2,85	1,64
Rfc1	P38630	5	5	2,45	1,37
Nmd3	P38861	11	11	3,28	3,01
Kog1	P38873	21	21	2,77	1,99
Tif4631	P39935	13	11	3,91	3,67

Snu13	P39990	7	7	2,74	2,02
Nug1	P40010	14	14	2,47	3,03
Nup82	P40368	18	18	4,15	4,96
Nup159	P40477	9	9	2,04	3,46
Rlp7	P40693	8	8	2,73	0,89
Nop2	P40991	13	13	2,21	2,46
Gpd2	P41911	5	4	2,11	1,56
Rtt101	P47050	7	7	2,63	2,57
Iml1	P47170	5	5	2,19	1,62
Nup84	P52891	5	5	2,35	1,59
Seh1	P53011	13	13	1,87	2,06
Ygr250c	P53316	6	6	2,59	1,94
Rpt4	P53549	12	11	2,53	0,76
Nog2	P53742	13	13	2,49	1,83
Imp4	P53941	6	6	1,96	1,92
Ssl2	Q00578	6	6	2,63	2,10
Ubp3	Q01477	14	14	3,27	1,75
Nup116	Q02630	9	9	3,93	3,73
Muk1	Q02866	25	25	2,28	1,00
Nog1	Q02892	23	23	2,00	4,07
Dig1	Q03063	13	13	2,05	1,95
Trs130	Q03660	7	7	2,58	1,38
Arx1	Q03862	4	4	2,33	1,58
Mtc5	Q03897	16	16	3,21	2,10
Mtq2	Q03920	20	20	3,99	5,97
Mms1	Q06211	12	12	3,29	3,19
Yll032C	Q07834	10	10	2,43	1,72
Dis3	Q08162	9	9	2,49	1,77
Crt10	Q08226	7	7	2,88	3,15
Yra1	Q12159	6	6	2,03	3,66
Rrp12	Q12754	15	15	3,61	1,31

mtq2-N122 versus TAP-alone					
**Gene names**	**Accession numbers**	**Peptides**	**Unique Peptides**	**-log$_{10}$ (p-value)**	**-log$_{2}$ (fold-change)**
**Enriched proteins**					
Sla1	P32790;A6ZKU1	14	14	4,76	5,72
Erb1	Q04660;A6ZMA9	13	13	1,96	1,77
Prt1	P06103;A6ZPJ1	21	21	2,08	2,63
Ald5	P40047;A6ZR27;P32872	24	23	2,89	1,58
Dcp2	P53550;A6ZRW5	10	10	2,74	2,37
Dbp2	P24783;A6ZRX0	16	15	1,90	1,88
Ypp1	P46951;A6ZUK8	7	7	2,63	1,91
Rpc82	P32349;A6ZX64	28	28	2,16	1,26
Dbp10	Q12389;A6ZXU0	3	3	1,98	1,42
Rps1A	P33442;C7GSL4;B5VNY0 B3RHV0;A7A1W1	21	4	2,16	1,38
Trp5	P00931	8	8	2,14	1,61
Tef1	P02994	39	39	2,14	0,99
Ths1	P04801	15	15	1,69	1,20
Sup35	P05453	22	22	3,11	1,66
Rpl9A;Rpl9B	P05738;P51401	9	9	2,63	1,52
Rps9B;Rps9A	P05755;O13516	13	13	1,93	1,94
Snf1	P06782	11	11	1,74	1,25
Ade3	P07245	9	7	2,16	0,98
Leu1	P07264	27	27	1,60	2,74
Bcy1	P07278	6	6	3,70	3,59
Ilv2	P07342	32	32	2,69	3,15
Rpc40	P07703	21	21	2,04	1,62
Gcd1	P09032	13	13	3,43	3,57
Mis1	P09440	38	36	2,54	1,98
Rps24B; Rps24A	P0CX32;P0CX31	7	7	2,39	1,13
Rps4B;Rps4A	P0CX36;P0CX35	18	18	1,98	1,19
Rps6B;Rps6A	P0CX38;P0CX37	10	10	2,56	1,34
Rna1	P11745	10	10	2,80	2,03
Sst2	P11972	15	15	3,68	2,51
Pdr1	P12383	27	27	3,08	2,71
Sup45	P12385	17	17	3,54	2,02
Tcp1	P12612	24	24	3,36	1,31
Lat1	P12695	25	25	2,03	1,32
Nat1	P12945	14	14	2,75	2,99
Ste12	P13574	13	13	3,44	2,33
Msi1	P13712	5	5	2,58	2,69
Yak1	P14680	44	44	1,80	1,76
Gfa1	P14742	44	44	1,83	2,28
Mss116	P15424	26	26	4,19	2,79

Trm1	P15565	10	10	2,23	1,26
Pdx1	P16451	23	23	1,79	1,70
Vma1	P17255	55	55	1,81	1,83
Pho81	P17442	16	16	3,13	3,13
Rnr1	P21524	43	31	2,31	1,87
Gas1	P22146	11	11	1,93	1,58
Ret1	P22276	44	44	1,94	2,03
Rfa1	P22336	10	10	1,64	1,32
Ssd1	P24276	28	28	2,99	2,48
Pho84	P25297	10	10	1,72	1,51
Ctr86	P25355	8	8	2,49	2,26
Cha1	P25379	22	22	1,97	2,04
Rps2	P25443	11	11	2,14	1,50
Pat1	P25644	10	10	3,44	2,92
Cdc48	P25694	35	35	2,15	0,75
Rpl5	P26321	10	10	3,07	1,16
Urk1	P27515	13	13	1,90	1,16
Mcm4	P30665	29	29	1,79	1,42
Nam7	P30771	45	45	3,24	2,53
Ccr4	P31384	7	7	2,27	1,34
Gcd6	P32501	27	27	2,37	3,43
Gcd7	P32502	9	9	2,92	3,24
Vph1	P32563	14	14	1,79	1,72
Ecm32	P32644	4	4	1,77	1,30
Fre1	P32791	5	5	2,73	2,30
Prs1	P32895	11	11	1,64	1,31
Rpt1	P33299	16	16	2,25	1,06
Sqt1	P35184	10	10	1,83	2,24
Dhr2	P36009	5	5	2,53	3,12
Mae1	P36013	14	14	2,07	2,28
Gly1	P37303	9	9	1,73	2,27
Grs1	P38088	20	20	1,86	1,48
Sea4	P38164	27	27	3,79	2,54
Nup170	P38181	32	32	1,96	1,80
Pby1	P38254	5	5	3,16	2,41
Enp1	P38333	10	10	2,27	2,35
Tps3	P38426	14	13	4,16	1,57
Rfc1	P38630	5	5	3,21	2,49
Yhr020W	P38708	34	34	2,29	2,23
Sfb3	P38810	13	13	1,76	1,03
Nmd3	P38861	11	11	4,04	3,71
Kog1	P38873	21	21	2,11	1,49
Hxt7;Hxt6	P39004;P39003	5	3	2,47	3,08
Cct4	P39078	23	23	2,54	1,68
Cct6	P39079	15	15	2,14	1,73

Ilv3	P39522	23	23	1,84	1,58
Tif4631	P39935	13	11	2,84	4,32
Snu13	P39990	7	7	3,10	2,47
Edc3	P39998	6	6	2,29	3,04
Gle2	P40066	3	3	3,32	2,11
Ccs1	P40202	13	13	1,86	3,59
Nup82	P40368	18	18	3,63	5,34
Cct5	P40413	25	25	2,53	1,99
Pog1	P40473;A6ZVF4	10	10	1,96	2,77
Slm1	P40485	9	9	1,70	1,03
Rlp7	P40693	8	8	1,89	1,44
Mkt1	P40850	9	9	2,50	0,45
Nop2	P40991	13	13	2,42	4,01
Gcd10	P41814	2	2	1,78	2,34
Cct7	P42943	17	17	1,65	0,93
Gcn20	P43535	20	20	1,85	1,61
Urb2	P47108	19	19	2,32	3,19
Npa3	P47122	3	3	3,18	2,67
Iml1	P47170	5	5	1,65	1,69
Bat2	P47176	8	7	2,73	2,26
Nup84	P52891	5	5	3,34	2,87
Seh1	P53011	13	13	2,17	2,04
Pil1	P53252	12	11	2,82	1,75
Pbp1	P53297	15	15	1,69	3,10
Ygr250c	P53316	6	6	2,83	2,81
Trm112	P53738	7	7	2,25	6,32
Bre5	P53741	6	6	3,04	2,63
Nog2	P53742	13	13	3,26	3,04
Cnm67	P53865	12	12	2,47	3,27
Imp4	P53941	6	6	1,64	1,70
Act1	P60010	21	21	2,27	0,98
Rho3	Q00245	5	5	1,85	1,71
Ssl2	Q00578	6	6	3,59	2,05
Cdc14	Q00684	12	12	1,91	2,22
Ubp3	Q01477	14	14	1,75	2,50
Npl3	Q01560	7	7	4,24	2,10
Rpt6	Q01939	12	12	1,66	1,11
Nup116	Q02630	9	9	2,89	4,13
Muk1	Q02866	25	25	1,99	1,79
Nog1	Q02892	23	23	3,49	5,37
Dig1	Q03063	13	13	2,07	2,56
Rmt2	Q03305	3	3	3,17	2,10
Trs130	Q03660	7	7	2,98	1,97
Nab6	Q03735	6	6	1,80	2,30
Bul2	Q03758	5	5	3,29	2,23

Arx1	Q03862	4	4	3,13	2,22
Mtc5	Q03897	16	16	3,98	3,18
Mtq2	Q03920	20	20	3,20	6,37
Hmo1	Q03973	5	5	1,73	3,75
Ty1AMr1; Ty1A-BI	Q04215;Q12266	19	1	1,80	2,43
Mms1	Q06211	12	12	2,98	2,82
Iki3	Q06706	11	11	2,73	2,22
Bre1	Q07457	47	47	1,74	1,11
Puf3	Q07807	3	3	3,55	2,66
Yll032c	Q07834	10	10	2,36	2,78
Noc3	Q07896	6	6	2,20	1,54
Dis3	Q08162	9	9	1,91	1,81
Brx1	Q08235	7	7	1,68	1,84
Gpb1	Q08886;REV_P21268	8	8	2,36	1,98
Rtt10	Q08924	12	12	3,24	2,66
Yol098C	Q12496	7	7	2,00	2,52
Rrp12	Q12754	15	15	3,05	1,94

Supplementary References

1. Tyanova, S., Temu, T. and Cox, J. (2016) The MaxQuant computational platform for mass spectrometry-based shotgun proteomics. *Nat Protoc*, **11**, 2301-2319.
2. Deutsch, E.W., Csordas, A., Sun, Z., Jarnuczak, A., Perez-Riverol, Y., Ternent, T., Campbell, D.S., Bernal-Llinares, M., Okuda, S., Kawano, S. *et al.* (2017) The ProteomeXchange consortium in 2017: supporting the cultural change in proteomics public data deposition. *Nucleic Acids Res*, **45**, D1100-D1106.
3. Mullineux, S.T. and Lafontaine, D.L. (2012) Mapping the cleavage sites on mammalian pre-rRNAs: where do we stand? *Biochimie*, **94**, 1521-1532.
4. Figaro, S., Scrima, N., Buckingham, R.H. and Heurgue-Hamard, V. (2008) HemK2 protein, encoded on human chromosome 21, methylates translation termination factor eRF1. *FEBS Lett*, **582**, 2352-2356.
5. Figaro, S., Wacheul, L., Schillewaert, S., Graille, M., Huvelle, E., Mongeard, R., Zorbas, C., Lafontaine, D.L. and Heurgue-Hamard, V. (2012) Trm112 is required for Bud23-mediated methylation of the 18S rRNA at position G1575. *Mol Cell Biol*, **32**, 2254-2267.
6. Wery, M., Ruidant, S., Schillewaert, S., Lepore, N. and Lafontaine, D.L. (2009) The nuclear poly(A) polymerase and Exosome cofactor Trf5 is recruited cotranscriptionally to nucleolar surveillance. *RNA*, **15**, 406-419.
7. Schillewaert, S., Wacheul, L., Lhomme, F. and Lafontaine, D.L. (2012) The evolutionarily conserved protein Las1 is required for pre-rRNA processing at both ends of ITS2. *Mol Cell Biol*, **32**, 430-444.
